# Data‐Driven Materials Research and Development for Functional Coatings

**DOI:** 10.1002/advs.202405262

**Published:** 2024-09-19

**Authors:** Kai Xu, Xuelian Xiao, Linjing Wang, Ming Lou, Fangming Wang, Changheng Li, Hui Ren, Xue Wang, Keke Chang

**Affiliations:** ^1^ Key Laboratory of Advanced Marine Materials Ningbo Institute of Materials Technology and Engineering Chinese Academy of Sciences Ningbo Zhejiang 315201 China; ^2^ Center of Materials Science and Optoelectronics Engineering University of Chinese Academy of Sciences Beijing 100049 China

**Keywords:** artificial intelligence, data‐driven, functional coatings, machine learning, materials design

## Abstract

Functional coatings, including organic and inorganic coatings, play a vital role in various industries by providing a protective layer and introducing unique functionalities. However, its design often involves time‐consuming experimentation with multiple materials and processing parameters. To overcome these limitations, data‐driven approaches are gaining traction in materials science. In this paper, recent advances in data‐driven materials research and development (R&D) for functional coatings, highlighting the importance, data sources, working processes, and applications of this paradigm are summarized. It is begun by discussing the challenges of traditional methods, then introduce typical data‐driven processes. It is demonstrated how data‐driven approaches enable the identification of correlations between input parameters and coating performance, thus allowing for efficient prediction and design. Furthermore, carefully selected case studies are presented across diverse industries that exemplify the effectiveness of data‐driven methods in accelerating the discovery of new functional coatings with tailored properties. Finally, the emerging research directions, involving integrating advanced techniques and data from different sources, are addressed. Overall, this review provides an overview of data‐driven materials R&D for functional coatings, shedding light on its potential and future developments.

## Graphical abstract

Functional coatings play a vital role in various industries for their protective and functional properties. However, its design often involves time‐consuming experimentation with multiple materials and processing parameters. To overcome these limitations, data‐driven approaches are gaining traction in materials science. This review provides an overview of data‐driven materials R&D for functional coatings, shedding light on its potential and future developments.

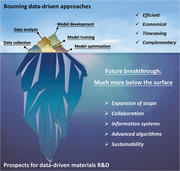

## 1 Introduction

**Figure 1:**
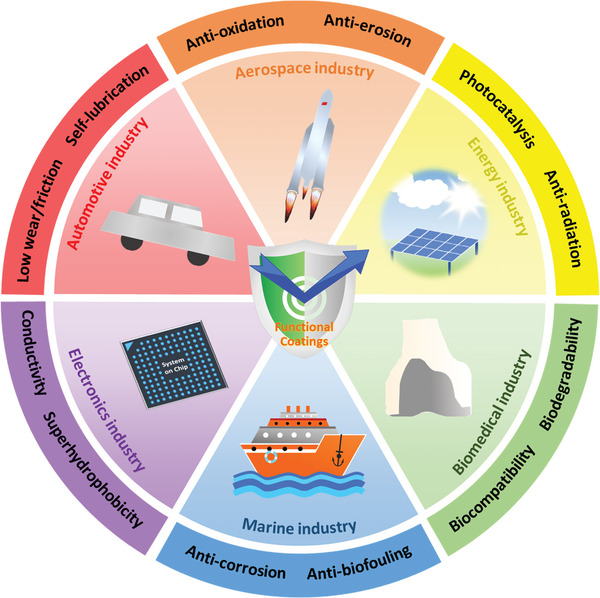
Specific protection or function needs of functional coatings in versatile industrial applications.

The tremendous development of high‐tech industry in the last decades has led to the actuality that the modern materials are increasingly served in harsh environments and are always in urgent needs of diversified functionalities. Functional coatings refer to a wide range of materials engineered to provide desirable surface properties, such as thermal barrier,^[^ ^1^, ^2^, ^3^, ^4^ ^]^ anticorrosion,^[^ ^5^, ^6^, ^7^ ^]^ wear‐resistance,^[^ ^8^, ^9^, ^10^, ^11^ ^]^ antifouling property,^[^ ^12^, ^13^, ^14^ ^]^ antimicrobial activity,^[^ ^15^, ^16^ ^]^ self‐healing capability,^[^ ^17^, ^18^, ^19^ ^]^ superhydrophobicity,^[^ ^20^, ^21^, ^22^ ^]^ conductivity,^[^ ^23^, ^24^, ^25^, ^26^ ^]^ color shift,^[^ ^27^, ^28^, ^29^ ^]^ and etc.^[^ ^30^, ^31^, ^32^, ^33^, ^34^, ^35^ ^]^ These coatings are an essential component of various industries that can fulfill specific requirements and advance the related material technology, ranging from aerospace and automotive to biomedical and electronics. The choice of customized coating depends on the specific protection or function needs in an industrial application, as show in **Figure** **1**.

- In the aerospace industry, functional coatings, such as thermal/environmental barrier coatings (T/EBCs) can protect aero‐engines components from high‐temperature exposure, enabling the engine to work at higher temperatures with consequently remarkable enhancement in efficiency and safety, bringing breakthrough in thrust‐to‐weight ratio, and reducing maintenance costs.^[^ ^1^, ^2^, ^3^, ^4^ ^]^ For the moving parts of spacecrafts operated in the low earth orbit, nanocomposite films such as MoS_2_‐based films can achieve high irradiation tolerance to atomic oxygen and self‐adaptive lubrication by manipulating the structural transformation of MoS_2_, doping with certain metals or compounds, introducing multilayer, and designing post‐treatments.^[^ ^36^, ^37^, ^38^ ^]^
- In the marine industry, anticorrosion coatings, such as epoxy, polyurethane, and zinc‐based coatings, act as a protective barrier on metal surfaces to prevent corrosion and even complex multifactor coupling damage from saltwater or deep‐sea exposure.^[^ ^6^, ^39^, ^40^ ^]^ Also, antifouling coatings, include self‐polishing copolymers and controlled depletion paints, prevent the buildup of organisms like algae and barnacles on the hulls of ships.^[^ ^12^, ^13^, ^14^ ^]^ The build‐up of ice on facility surfaces can be prevented by anti‐icing coatings, leading to better performance and safety during services.^[^ ^20^, ^41^, ^42^ ^]^
- In the energy industry, the transition to cleaner and low‐carbon energy is high‐profile and irresistible. Photocatalytic coatings can be applied to solar panels to enhance their efficiency by converting more sunlight into electricity.^[^ ^43^, ^44^, ^45^ ^]^ These coatings can also be used to harness solar energy for water splitting, enabling the generation of clean and renewable hydrogen fuel.^[^ ^46^, ^47^ ^]^ Nuclear energy is also become an increasing crucial alternative for clean electrical power. Different protective coatings designed for accident‐tolerant fuel cladding materials can constantly withstand neutron irradiation, as well as high‐temperature and frictional wear.^[^ ^48^, ^49^, ^50^ ^]^
- In the automotive industry, functional coatings can improve the longevity of different parts and minimize material waste. For example, scratch‐resistant coatings protect car surfaces from damage, while coatings that reduce friction assist in the production of more fuel‐efficient engines.^[^ ^51^, ^52^ ^]^ Wear resistant coatings deposited on the surfaces of engine or moving components exhibiting low friction and wear under various operating conditions can help to decrease fuel consumption and prolong service life.^[^ ^53^, ^54^ ^]^
- In the biomedical industry, surface modification technology is indispensable for the long‐term application of medical implants or stents, as well as those nonimplanted medical devices, which can improve the biocompatibility, safety, and efficacy of biomaterials.^[^ ^23^, ^55^, ^56^ ^]^ Coatings designed to prevent bacterial attachment can reduce the risk of infections, while coatings that promote specific cellular activities can enhance tissue growth and recovery.^[^ ^57^, ^58^, ^59^ ^]^
- In the electronics industry, functional coatings can protect delicate circuitry from complex environmental damage and add desirable aesthetic properties. For example, anticorrosion or antidust coatings can reduce the risk of failure in electronic hardware or remove dust and debris on top of interdigitated electrodes,^[^ ^60^, ^61^ ^]^ while optical or electromagnetic coatings can improve the display quality or inspire specific physical characteristics of electronic devices.^[^ ^62^, ^63^, ^64^ ^]^ Additionally, with the growing interest in flexible and wearable sensing electronics, versatile functional coatings, including but not limited to electrically conductive coatings, superhydrophobic smart coatings, and low‐cost stretchable coatings, are explored and developed to fulfill the innovation demands in modern electronics.^[^ ^65^, ^66^, ^67^ ^]^

The growing demand for high‐performing and sustainable products has led to an increased interest in functional coatings. Despite their widespread applications, the R&D of innovative functional coatings remain a challenging task. Traditional methods used for the discovery of functional coatings with tailored properties rely heavily on trial‐and‐error, which have proved to be both costly and time‐consuming, particularly when dealing with complex coating systems. These systems often involve multiple components, phases, microstructures, properties, and preparation techniques, further exacerbating the challenges faced during the discovery process.

**Figure 2:**
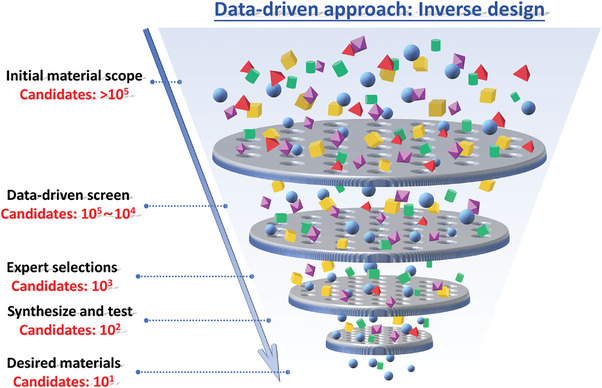
Workflow of inverse design for functional coatings from the ocean of data to desired materials by data‐driven approach.^[^ ^70^ ^]^

The ongoing development and interdisciplinary collaboration of computer technology and other fields, such as materials science in recent years have sparked significant interest in scientific data as the heart of state‐of‐the‐art research, current hardware and software enable the utilization of data at an unprecedented scale.^[^ ^68^, ^69^, ^70^, ^71^ ^]^ With vast amount of data now available in materials science and engineering, data‐driven approach has emerged as a promising choice showing great potential in reducing the time and cost for materials discovery.^[^ ^72^, ^73^, ^74^, ^75^, ^76^ ^]^ The discovery of highly tailored coatings utilizing machine learning (ML) can be treated as a tool within inverse design strategy, which is unlike the traditional diverse one, as shown in **Figure** **2**.^[^ ^70^ ^]^ The accumulated data can perform as a foundation for investigation, helping to reduce cost and speed up the development cycle of novel functional coatings by shrinking the search space toward the most promising regions.

Herein, the purpose of this review paper is to introduce the use of data‐driven techniques in the search for promising functional coatings, and to discuss the potential benefits and limitations of this approach. On this basis, we carefully discuss the inevitable challenges in traditional methods and the recent progress in data‐driven R&D for functional coatings. Moreover, we prospect the development trend of data‐driven approaches in future.

## 2 Traditional Methods of Coating Material Investigation

**Figure 3:**
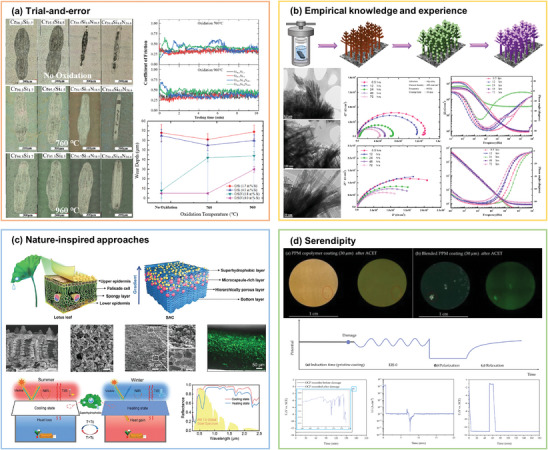
Traditional materials discovery approaches, including a) trial‐and‐error. Reproduced with permission.^[^ ^77^ ^]^ Copyright 2020, Elsevier. b) Empirical knowledge and experience. Reproduced with permission.^[^ ^78^ ^]^ Copyright 2023, Elsevier. c) Nature‐inspiration approaches. Reproduced with permission.^[^ ^79^ ^]^ Copyright 2024, John Wiley and Sons. d) Serendipity. Reproduced under the terms of the CC‐BY license.^[^ ^80^ ^]^ Copyright 2022, the Authors, published by MDPI, Basel, Switzerland.

Traditionally, materials to be used in functional coatings have been developed through the trial‐and‐error method. Different raw materials and chemical compounds are mixed together to make samples through specific processes Their properties are experimentally evaluated, such as thermal stability, hardness, toughness, chemical resistance, and electronic properties. Then, based on the performance, the composition and process are adjusted until the desired performance specifications are achieved. This process is lengthy and expensive, often requiring significant R&D resources. Some classic traditional material development strategies are shown in **Figure** **3**, including trial‐and‐error, empirical knowledge and experience, nature‐inspired, and serendipity. The following section briefly describes these traditional methods of materials investigation.

### 2.1 Trial‐and‐Error

Before the development of modern science, trial‐and‐error methods dominated materials science and chemistry. Researchers typically selected a set of possible material compositions or processing parameters and then tested these choices experimentally, with repeated experiments and refinements to determine the optimal combination of materials and properties. He et al.^[^ ^77^ ^]^ prepared two types of CrSi and CrSiN coatings with different Si content ratios by multiarc ion plating. It was found that the friction and wear performance of the CrSi‐based coatings with a high Si content was superior to that of the CrSi‐based coatings with a low Si content, but inferior to CrSiN coatings. In practice, there is tremendous complexity in the combination of material composition and processing technology. The approach is highly dependent on personal experience and intuition, making it difficult to comprehensively and systematically explore the optimal combination of materials. As a result, it can take a lot of time and resources to find an effective material solution.

### 2.2 Empirical Knowledge and Experience

Materials scientists often rely on pre‐existing expertise and experience to guide the materials development process. By analyzing existing materials science knowledge and experience, combined with multidisciplinary field crossover, it can help researchers to make the right decisions and thus improve the efficiency of materials science research. Chaouiki et al.^[^ ^78^ ^]^ combined the knowledge in the fields of electronic modulation and architectural engineering to grow a multifunctional coating with 3D fir tree structure on magnesium alloy matrix, upgrading the structure of the coating and improving the electrochemical efficiency. Although the method of combining experience and knowledge can eliminate a large number of useless attempts in advance, it has higher professional requirements for researchers.

### 2.3 Nature‐Inspired Approaches

The design and development of biomimetic materials are an important research direction in materials science. There are many high‐performance materials in nature with unique structures, and these natural materials have evolved over time to exhibit amazing properties, such as high strength, high toughness, self‐healing, and smart response. Researchers delve into the study of natural material structures and their formation processes, drawing inspiration from nature to develop novel materials through biomimetic design and bio‐inspired approaches to meet the needs of modern industrial and technological development. In nature, for example, the complex structure of plant leaves gives them excellent light absorption and wettability. Inspired by the longitudinal gradient characteristics and surface micro‐nanostructures of lotus leaves, the teams of Wang and Tao^[^ ^79^ ^]^ collaborated in the research to prepare a multifunctional thermoregulatory coating that integrated self‐cleaning of superhydrophobic surfaces and automatic adjustable solar reflectivity, and effective utilization of thermal infrared emission through a one‐step phase separation process. In this way, radiation refrigeration in high temperature environment and solar heating in low temperature environment were realized, thereby, achieving the Intelligent thermoregulation of “warm in winter and cool in summer.”

### 2.4 Serendipity

Occasionally, new materials are discovered by chance during unrelated experiments or observations. A team from ETH Zurich^[^ ^80^ ^]^ has discovered a polymer coating called polyphenylene propylene methacrylate (PPM) with self‐healing. This material is able to repair itself after being damaged, thus restoring its original protective properties. Initially, the researchers only found that the material had good anticorrosion properties, and then accidentally discovered its self‐healing properties. The emergence of such new substances or materials could open new horizons for the entire science community, which is of significant value but less likely to be duplicated.

Traditional experimental approaches for materials discovery have typically relied on trial‐and‐error methods. While these methods have been effective in the past, they have had some notable limitations, notably in terms of high costs and inefficiencies, especially in novel material systems with multiple components and phases. These methods are usually limited to testing of known materials and do not guarantee the discovery of new materials or properties, which directly limits the development and application optimization of materials science. In addition, these methods of materials development rely heavily on the experience and intuition of researchers, which requires a high level of researchers' own knowledge base and experience. Moreover, the preparation and testing conditions for each new material may be different, which makes it difficult to standardize the R&D process, and increase the difficulty of translating from the laboratory to real‐world applications.

In the face of these challenges, data‐driven approaches show great potential to significantly accelerate the discovery process of new materials, reducing the number of trials, and improving the accuracy of predictions by utilizing advanced algorithms and big data techniques. These methods can quickly identify and recommend potentially valuable material combinations within large chemical and material databases to optimize material performance and functionality. At the same time, data‐driven methods improve theory formation and scientific advancement by modeling more accurate correlations between material composition and properties, which is especially critical for the development of multifunctional coatings and high‐performance materials. The data‐driven approaches will be cover in detail in the following sections.

## 3 Data‐Driven Approaches

Data‐driven materials research and development is a research approach that involves the analysis of large sets of data from various sources to develop predictive models that can accurately predict the properties of a material before it is synthesized or fabricated in a laboratory. In the 1980s, the rise of ML and artificial intelligence (AI) catalyzed the application of data‐driven models to complex problems, leading to the development of early data‐driven frameworks. Entering the 21st century, the advent of big data technologies made it possible to process and analyze vast amounts of data, marking humanity's entry into the Industry 4.0 era. During this period, data‐driven approaches rapidly advanced, becoming one of the primary drivers of innovation.^[^ ^81^ ^]^ Composition and structure design, process modeling and optimization, and performance improvement are critical issues in the field of coating engineering, yet the inherent nonlinear processes within coatings present challenges that conventional research methods struggle to address. ML and deep learning (DL) have proven highly effective in handling these nonlinear problems in a data‐driven manner. As early as 2007, Barletta et al. utilized artificial neural network (ANN) technology to model electrostatic fluidized beds (EFB) and screened for the optimal NN model.^[^ ^82^ ^]^ In 2014, they applied new ML techniques, using support vector machines (SVM) to model the EFB coating process, achieving favorable predictive results.^[^ ^83^ ^]^ Currently, an increasing number of ML techniques, such as ANN and SVM, alongside Bayesian optimization and genetic algorithms, are being employed in functional coatings, often involving a combination of various methods for comparative use.^[^ ^84^ ^]^

### 3.1 Process Guideline

**Figure 4:**
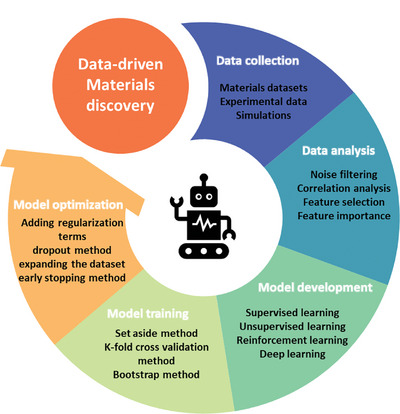
Framework of data‐driven approaches.

Leveraging the capabilities of extensive and diverse datasets, data‐driven approaches employ sophisticated modeling techniques to discern significant patterns, which can offer the potential to uncover hidden trends, and predict serving behaviors, and even optimize processes with unprecedented accuracy. The following process outlines the framework of data‐driven approaches (**Figure** **4**).

At first, collecting extensive materials datasets according to a determined data analysis framework. These datasets can come from various sources, including experimental data, materials databases, and simulations. The collected data needs to ensure its accuracy and completeness. It will be analyzed to identify correlations between the materials properties and potential performance. Correlation analysis can provide a statistical basis for the feature selection and validate whether the highly ranked features are coincident with generally accepted mechanisms, such as Maximum Information Coefficient (MIC), Pearson Correlation Coefficient (PCC). Models will be developed to predict the materials structures and properties based on the analyzed data. Depending on the type of task, these models can be built based on supervised learning, unsupervised learning, or reinforcement learning algorithms.

The developed models must be trained using existence data to make models reliable and accurate. Usually, the data are divided into two parts, a training set and a testing set, to train and test the model. There are several commonly used partitioning methods, such as the set aside method, the k‐fold cross validation method, and the bootstrap method.

While training, the developed models can be optimized by adjusting model parameters or adding additional data. Overfitting is a common issue, which refers to the phenomenon where a model has a small error on the training set data, but a large error on data samples outside the training set. Common solutions include the dropout method, L1 and L2 regularization, and the early stopping method. At last, predictions are made based on the optimized model to either improve existing materials or discover new materials.

### 3.2 Data Collection

There are several data sources that can be used for coatings development using data‐driven approaches in functional coatings development, commonly used mainly for the following categories: i) Experimental materials databases (Springer Materials, Cambridge Structural Database (CSD), Inorganic Crystal Structure Database (ICSD), National Institute of Standards and Technology (NIST) database, and Crystallography Open Database (COD)); ii) Computational materials databases (Materials Project, Automatic‐FLOW, Open Quantum Materials Database (OQMD), and Novel Materials Discovery (NOMAD) project); iii) Research articles and scientific literature;^[^ ^85^, ^86^, ^87^ ^]^ (iv) Patents and intellectual property databases (United States Patent and Trademark Office (USPTO), European Patent Office (EPO), World Intellectual Property Organization (WIPO)).

The integration of large amounts of data from different sources enables assistance in the identification of promising materials and processing parameters, minimizes both time and risk inherent in screening new materials. Consequently, the following section delineates approaches for the concrete implementation of data collation. In accordance with the insights derived from literatures, these data collation methodologies are systematically classified into several categories, namely, high‐throughput experimentation, computational modeling, data mining, and big data.

#### 3.2.1 High‐Throughput Experimentation

In recent decades, researchers are working with the principles of multicomponent composition gradient and multiple samples at once, and have gradually proposed and conducted high‐throughput preparation or characterization methods in discovering new materials such as alloys, polymers, inorganics, etc.^[^ ^88^, ^89^, ^90^, ^91^, ^92^ ^]^ In the realm of high‐throughput experiments, several critical factors should be considered, including automated instrumentation and processes, parallel processing, as well as data management and analysis. Automation, as applied comprehensively throughout the experimental workflow, spans domains such as sample synthesis and measurement. This pervasive automation serves the pivotal role of minimizing manual intervention, thereby mitigating the potential for inadvertent errors. Parallel processing, an integral component of high‐throughput experimentation, expedites research endeavors through the simultaneous execution of multiple experiments. This parallelization significantly accelerates the process of data generation and analysis.

**Figure 5:**
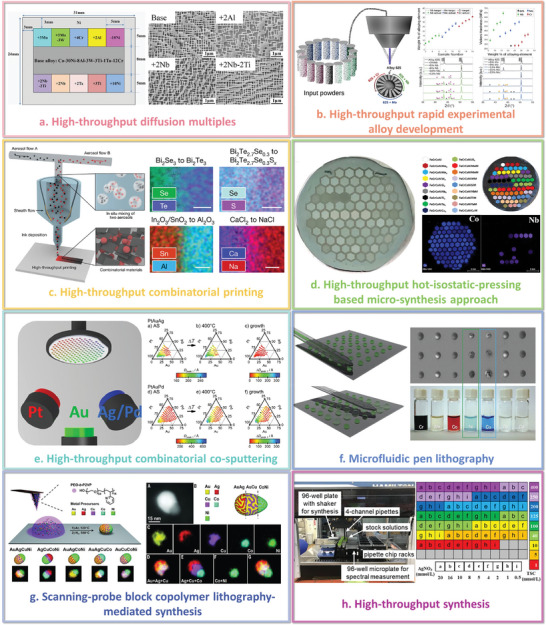
Summary of typical high‐throughput experimental preparation methods. a) Effective alloy design of high‐Cr CoNi‐based superalloys via multicomponent high‐throughput diffusion multiples. Reproduced with permission.^[^ ^95^ ^]^ Copyright 2023, Elsevier. b) Modifying Alloy 625 based on the integrated high‐throughput rapid experimental alloy development (HT‐READ) methodology. Reproduced with permission.^[^ ^96^ ^]^ Copyright 2021, Elsevier. c) The development of combinatorial materials from aerosols by high‐throughput combinatorial printing (HTCP). Reproduced under the terms of the CC‐BY license.^[^ ^97^ ^]^ Copyright 2023, the Authors, published by Springer Nature. d) The exploration of CoCrFeNi‐based high entropy alloys enabled by the newly developed high‐throughput hot‐isostatic‐pressing based microsynthesis approach (HT‐HIP‐MSA). Reproduced with permission.^[^ ^98^ ^]^ Copyright 2022, Elsevier. e) Study of thermal stability in ternary Pt‐Au‐Ag/Pd nanocrystalline alloys using high‐throughput combinatorial cosputtering. Reproduced with permission.^[^ ^102^ ^]^ Copyright 2020, Elsevier. f) Femtolitre chemistry of screening new metal‐peptide networks assisted by microfluidic pen lithography (MPL). Reproduced with permission.^[^ ^104^ ^]^ Copyright 2013, Springer Nature. g) A five‐element library (Au, Ag, Cu, Co, and Ni) of unary and multimetallic nanoparticles made via the scanning‐probe block copolymer lithography (SPBCL) mediated synthesis technique. Reproduced with permission.^[^ ^106^ ^]^ Copyright 2016, American Association for the Advancement of Science. h) High‐throughput synthesis of silver nanoplates in 96‐well plates by mixing the stock solutions at room temperature with an automated liquid handler. Reproduced with permission.^[^ ^110^ ^]^ Copyright 2022, Elsevier.

In reality, a number of high‐throughput experimental preparation methods obeying different principles have been designed in different fields, which include but not limited to the high‐throughput diffusion multiples,^[^ ^93^, ^94^, ^95^ ^]^ high‐throughput rapid experimental alloy development (HT‐READ) methodology,^[^ ^96^ ^]^ high‐throughput combinatorial printing (HTCP),^[^ ^97^ ^]^ high‐throughput hot‐isostatic‐pressing based microsynthesis approach (HT‐HIP‐MSA),^[^ ^98^ ^]^ high‐throughput combinatorial co‐sputtering,^[^ ^99^, ^100^, ^101^, ^102^ ^]^ microfluidic pen lithography (MPL),^[^ ^103^, ^104^, ^105^ ^]^ scanning‐probe block copolymer lithography (SPBCL) mediated synthesis technique,^[^ ^106^, ^107^, ^108^, ^109^ ^]^ and other high‐throughput synthesis approach,^[^ ^110^, ^111^ ^]^ as summarized in **Figure** **5**. These laboratory techniques have been used successfully in the materials discovery, which can be used to quickly screen a large number of materials for a particular property. By screening a large number of materials simultaneously, researchers can identify promising candidates for further optimization. The researchers have combined design of experiment (DoE) with high‐throughput experiments to statistically analyze the effects of different factors on the performance of the materials. The method will further improve the efficiency of high‐throughput experiments, thus obtaining more critical experimental data and improving the progress of the screening process.^[^ ^112^, ^113^ ^]^

The prodigious volume of data generated during these experiments necessitates specialized methodologies for data management and analysis. These approaches are essential for discerning implicit patterns within the data, which, in turn, serve as reference points for subsequent investigations.

#### 3.2.2 Computational Modeling

**Figure 6:**
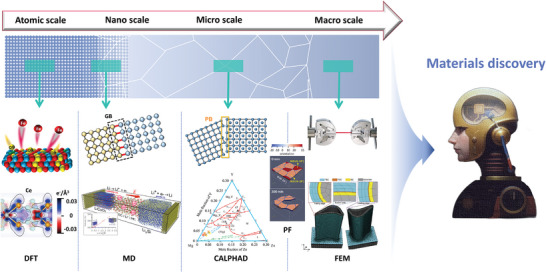
Illustration of diverse scale of computational modeling. Typical methods from the scale of atoms and electron to macroscale are density functional theory (DFT), molecular dynamics (MD), calculation of phase diagrams (CALPHAD), phase‐field (PF) modeling, and finite element modeling (FEM). Reproduced with permission.^[^ ^116^ ^]^ Copyright 2023, Elsevier. Reproduced under the terms of the CC‐BY license.^[^ ^117^ ^]^ Copyright 2023, the Authors, published by Springer Nature. Reproduced with permission.^[^ ^118^ ^]^ Copyright 2020, Elsevier. Reproduced with permission.^[^ ^119^ ^]^ Copyright 2017, Springer Nature. Reproduced under the terms of the CC‐BY license.^[^ ^120^ ^]^ Copyright 2020, the Authors, published by American Association for the Advancement of Science. Reproduced with permission.^[^ ^121^ ^]^ Copyright 2021, Elsevier. Reproduced with permission.^[^ ^122^ ^]^ Copyright 2022, Elsevier. Reproduced with permission.^[^ ^123^ ^]^ Copyright 2014, Elsevier.

Computational modeling methods have emerged as powerful tools for materials discovery.^[^ ^114^, ^115^ ^]^ These computational methods can significantly reduce the need for experimental trial‐and‐error, and provide insights into the underlying mechanisms that govern materials properties. Each method has its own strengths and limitations, and researchers often utilize a combination of these techniques to gain a comprehensive understanding of material properties and behaviors. **Figure** **6** displays commonly used computational modeling methods in materials discovery at different scales:

- Density functional theory (DFT): DFT is a widely used method for investigating the electronic structure of materials from the viewpoint of atoms and electrons.^[^ ^124^, ^125^ ^]^ It enables prediction of various properties, such as band structure, defect formation energies, and reaction energies, providing valuable insights into the behavior of materials. DFT provides data for machine learning including electronic structures, band structures, electrochemical, and thermodynamic properties.^[^ ^87^, ^126^ ^]^
- Molecular dynamics (MD): MD simulations provide a dynamic view of material behavior under various conditions, such as phase transitions and mechanical stresses, by numerically solving Newton's equations of motion. These simulations require precise initial specifications, including the types and quantities of molecules, their spatial coordinates, and initial velocities, to generate data on the positions and velocities of atoms at successive time steps. For instance, Xiao et al.^[^ ^127^ ^]^ used MD simulations to investigate the formation of a biomimetic nanostructured ultrablack coating, revealing how molecular interactions at the nanoscale influence material properties. This study can provide machine learning with time‐series data on atomic positions and velocities.
- Calculation of phase diagrams (CALPHAD): The CALPHAD approach is a computational method for calculating phase diagrams of multicomponent systems, which allows one to design target microstructure and optimize macro property.^[^ ^128^, ^129^, ^130^, ^131^ ^]^ These calculations provide machine learning with data on phase stability, transition points, and compositional relationships. For instance, The Chang's team conducted an in‐depth study on the alloy coatings. They calculated the effect of Si addition on the mechanical properties of NbTiZr refractory medium‐entropy alloys (RMEA) by the CALPHAD method.^[^ ^132^ ^]^ This data can train models to predict how alloy hardness, toughness, and strength change with different Si contents. Similarly, the thermodynamic and kinetic data from the study of Lou et al.^[^ ^133^ ^]^ on the oxidation behavior of TiN system coatings with refractory metal elements can be utilized by machine learning models. The trained model can be used to predict the oxidation behavior and corrosion resistance of coatings under different environmental conditions (such as temperature and oxygen partial pressure).
- Phase‐field (PF) modeling: It is used to study phase transformations, grain growth phenomenon, fracture behavior, and crack propagation in materials.^[^ ^134^, ^135^ ^]^ Dynamic equations governing the evolution of PF variables are derived via the variational principle, elucidating how these variables change over time. Initial distribution of PF variables at the inception of the simulation is determined, then, considerations are made regarding the behaviors at boundaries. External driving forces, such as temperature gradients or chemical gradients, are introduced into the model, influencing both the direction and rate of phase field evolution. Gao et al.^[^ ^122^ ^]^ utilized PF modeling to reveal the mechanisms of grooving and agglomeration in polycrystalline NiSi thin films on monocrystal Si substrates, offering insights into the dewetting phenomena of the polycrystalline thin film. PF modeling results can be used for machine learning models to predict the behavior of materials under different environmental conditions and optimize material design and processing techniques.
- Finite element modeling (FEM): FEM is a numerical analysis method that approximates the solution of the whole problem by partitioning a complex continuous problem into simple subregions and performing numerical computations on the subregions in sequence. It is widely used in macroscale structural analysis and various engineering applications.^[^ ^136^ ^]^ Sajadi et al.^[^ ^137^ ^]^ used FEM to develop a continuous plastic damage model to understand the damage expansion law of conformal coatings under compressive loads. This approach can provide machine learning with detailed data on structural stresses, deformations, material behaviors, etc. Machine learning models can leverage this data to predict and optimize material and structural performance under complex loading conditions, thereby improving design and material selection.

#### 3.2.3 Data Mining and Big Data

The rise of high‐performance computing platforms, cloud computing and IoT sensors infrastructure, has resulted in the generation of vast amounts of data, opening new avenues for materials discovery processes. Data mining and big data technologies play a crucial role in deciphering and utilizing this plethora of information to enhance materials development. Data mining involves the extraction of previously unknown and potentially useful patterns from large datasets and applying computational and statistical technology to data from experiments and simulations. It serves to unveil intricate relationships between material components, experimental conditions, and material properties. By distilling the most relevant features from high‐dimensional datasets, identifying errors, anomalies, or unanticipated outcomes within experimental datasets, and utilizing techniques such as classification, clustering, and association rules, data mining provides invaluable guidance to researchers in optimizing research efforts and potentially unearthing unnoticed phenomena. Big data, characterized by its vast volume, multifaceted composition, and real‐time attributes, encompasses large and diverse sets of data on materials, which can be used to train ML algorithms to identify critical properties of coating materials. However, big data often arrives in an unfiltered or unprocessed state, laden with noise and anomalies. Specialized tools and methodologies are required to preprocess and refine this data, rendering it amenable for practical utilization. Despite its challenges, big data has emerged as a formidable instrument in the fields of materials design and analysis. Together, the combination of data mining and big data technologies facilitates a deeper understanding of the intricate interplay of variables in materials development and enables the identification of critical properties of materials, thereby optimizing research efforts and leading to the development of new functional coatings. For example, Nikalin et al.^[^ ^138^ ^]^ reviewed the patent literature of the past 15 years dealing with waterborne coatings used for painting metal surfaces and concluded concerning the most promising and demanded areas in the waterborne paint and varnish sector. Momber et al.^[^ ^85^, ^86^ ^]^ proposed a concept of a fully data‐based procedure for the condition monitoring of protective coating systems of large onshore wind turbines. This data and methodology can provide machine learning with critical information on material characteristics, performance prediction, behavior pattern identification, and new material discovery.

### 3.3 Data Analysis

#### 3.3.1 Data Preprocessing and Feature Engineering

In the process of data analysis and model building, data preprocessing is the first essential step to ensure the quality and consistency of data, which is foundational for subsequent analyses. Data preprocessing mainly includes data cleaning and data transformation. Data cleaning involves handling missing values, anomalies, and obvious erroneous data. Common methods for dealing with missing values include linear and polynomial interpolation. Linear interpolation estimates missing data based on the values of adjacent points and is suitable for cases where data changes are relatively stable; polynomial interpolation is used when data changes are more complex, providing a more accurate interpolation. Anomaly handling often involves identifying and correcting or removing values that significantly differ from others, typically accomplished through statistical methods like box plots or Z‐score methods.

Data standardization and normalization are important parts of data transformation. Standardization is achieved by calculating the Z‐score of the data, with the formula

(1) $$Z\;=\frac{\left(X-\mu \right)}{\sigma }\;$$

where μ and σ represent the mean and standard deviation of the data, respectively. This method normalizes the data to have zero mean and unit variance, helping to eliminate the effects of different scales. Normalization adjusts the data range to [0,1] using the formula

(2) $$\;X_{\mathrm{norm}}=\frac{\left(X-X_{\min}\right)}{\left(X_{\max}-X_{\min}\right)}$$

where *X* represents the original data points, with *X*_min_​ and *X*_max_​ being the minimum and maximum values in the dataset, respectively. It makes the data comparable on the same scale and suitable for input into most machine learning algorithms.

After data preprocessing, feature engineering is conducted to extract and construct features that aid in model prediction. This includes creating new statistical parameters, performing effective variable selection, and necessary data format transformations to meet the model's needs. For example, calculating the moving average of time series data to smooth the data or constructing composite features (such as interaction terms) to increase the model's nonlinear capabilities and interpretability. This step not only enhances the model's predictive power but also aids in improving the model's understanding and interpretation of the data.

#### 3.3.2 Feature Screening

In the preparatory phase of model training, comprehensive data analysis is essential to maximize the utility of data and ensure accurate model predictions. The process includes several critical, nonsequential steps that independently contribute to understanding and preparing the data for modeling. Correlation analysis is initiated using statistical methods (PCC and MIC) to evaluate both linear and nonlinear relationships between features and the target variable. The PCC, which measures the linear association between variables, is calculated as

(3) $$PCC\;=\frac{\sum \left(x_{i}-\bar{x}\right)\left(y_{i}-\bar{y}\right)}{\sqrt{\sum {\left(x_{i}-\bar{x}\right)}^{2}{\left(y_{i}-\bar{y}\right)}^{2}}}\;$$

where *x*_i_ and *y*_i_ are the individual sample points, and $\bar{x}$ and *y* are the mean values of the variables.

Advanced statistical techniques such as Principal Component Analysis (PCA) and Factor Analysis are employed to delve deeper into the data structure and reduce its dimensionality. PCA, for example, transforms high‐dimensional data into a lower‐dimensional space by identifying principal components that capture the most variance

(4) $$Z\;=\;X-\bar{X}$$

(5) $$PCA\left(Z\right)\;=\;ZW$$

where *Z* represents the data adjusted by subtracting the mean $\bar{X}$, and *W* is the matrix of eigenvectors derived from the covariance matrix of *Z*.

Feature importance analysis plays a pivotal role in the development of machine learning models, particularly when employing complex algorithms such as Random Forests (RF) and Gradient Boosting (GB). These algorithms come equipped with the ability to assess the contribution of each feature toward the model's predictive performance, quantifying the specific impact of each feature on model performance.

In RF, feature importance is calculated by observing the contribution of each feature to reducing model error during the construction of decision trees. Specifically, it considers the contribution of each feature in reducing impurity (such as Gini impurity or entropy) when used as a split node across all trees. A higher contribution indicates that the feature significantly impacts model prediction accuracy.

GB assess feature importance slightly differently. They typically consider the contribution of each feature to performance improvement in each iteration. If a feature is frequently used to split nodes across multiple iterations and each split significantly enhances the model's performance, the feature is considered important. It is a crucial step in ensuring that the model is ready to efficiently handle complex learning tasks. It provides a solid foundation for model training, ensuring the accuracy and reliability of predictions.

### 3.4 Machine Learning (ML)

The fundamental tenets of ML entail the discernment of regulations and patterns from specific datasets through computational means. Within this process, automated enhancement stands as a distinguishing feature of ML algorithms, endowing them with the capacity to iteratively refine their performance through critically assessment of datasets without human intervention. Through the iterative optimization of models over multiple cycles, ML algorithms consistently elevate both accuracy and holistic efficacy, thereby encouraging the model within a state of refinement.

**Figure 7:**
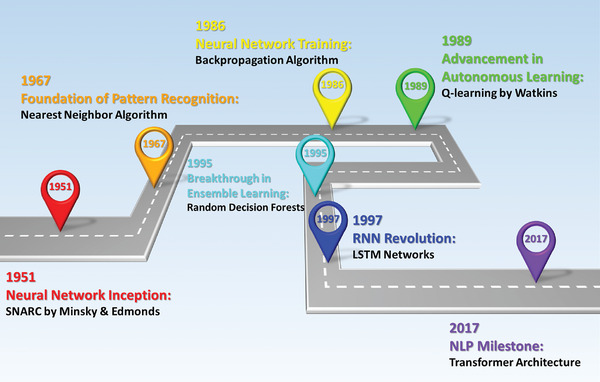
A brief timeline of key milestones for the development of ML.

**Figure** **7** highlights the chronological trajectory of algorithmic discoveries that have wielded a substantial influence within the realm of ML. The genesis of ML traces back to the 1950s and 1960s, embarking on a journey characterized by significant developmental epochs that spanned from the inception of NN algorithms to the emergence of deep learning. Throughout this evolutionary trajectory, foundational algorithms, and concepts have been successively introduced.

**Figure 8:**
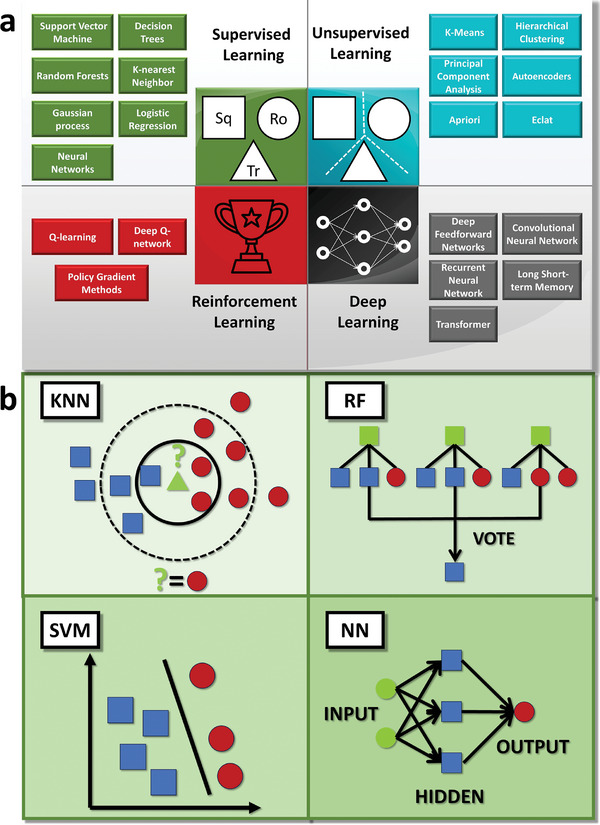
a) Common machine learning (ML) algorithms organized into four primary categories. b) A concise schematic illustrating four prominent ML algorithms commonly used in supervised learning for materials discovery: K‐nearest neighbors (KNN), random forest (RF), support vector machine (SVM), and neural networks (NN).

Inspired from the timeline of key milestones, we provide a visual representation of commonly employed ML algorithms in **Figure** **8**a. It is always organized into four primary categories: supervised learning, unsupervised learning, reinforcement learning, and deep learning. In supervised learning, algorithms endeavor to discern the relationship between input parameters and corresponding output parameters, with access to labeled output data. Logistic regression, a supervised learning technique, is apt for binary classification and finds utility in fault detection. Gaussian processes not only yield predicted values but also provide confidence intervals for each prediction. Conversely, unsupervised learning operates without the explicit pursuit of predefined objectives, instead aiming to uncover inherent structures within a given dataset, which primarily serves classification and clustering purposes. Reinforcement learning, conversely, serves to optimize process parameters through iterative adjustments guided by feedback from human settings. Deep learning is especially valuable in image recognition and processing, notably in the context of coatings. Among these, supervised learning finds extensive application in data processing. Figure 8b presents a schematic diagram of four prominent algorithms commonly used in supervised learning for materials discovery, which are K‐nearest neighbors (KNN), RF, SVM, and NN.

KNN is an instance‐based learning algorithm that operates by identifying the k nearest data points in proximity to a given sample, leveraging the labels of these nearest neighbors to predict the label of new data points. It addresses both regression and classification problems by computing distances between the samples to be classified and known samples. The distance is defined as follows

(6) $$D\;\left(x,x_{i}\right)=\sqrt{\sum_{j}{\left(x_{i}-x_{ij}\right)}^{2}}$$

where *D*(*x*, *x_i_*) is the distance between data point *x* and training sample *x_i_*. This is called Euclidean distance.

KNN boasts simplicity and ease of implementation, however, its computational demands become formidable when dealing with extensive datasets. This stems from the necessity to compute distances between each point and all other points, with the computational burden intensifying as data dimensionality increases.

RF, in contrast, represents an ensemble learning method that derives predictions from a consensus of multiple decision trees. A single decision tree can exhibit excessive branching, leading to poor generalization and overfitting on training data. By aggregating the outputs of multiple decision trees, RF enhances generalization while mitigating overfitting. Its core process involves constructing numerous decision trees and combining their predictions through voting or averaging.

SVM is a binary classification model, which seeks to find a hyperplane that maximizes the margin between two classes within a dataset. For linearly separable data, the objective is to identify a decision boundary

(7) $$w^{T}x_{i}+b\;=\;0$$

satisfying the equation

(8) $$y_{i}\left(w^{T}x_{i}+b\right)\geq 1,\forall i$$

where *y_i_* represents the label of *x_i_*. When dealing with nonlinearly separable data, a mapping to a higher‐dimensional space facilitates the discovery of a linear hyperplane in this expanded context.

NN, inspired by the human nervous system, comprises interconnected artificial neurons arranged in layered structures, emulating the neural processes in the human brain. The NN's formulation is as follows

(9) $$y\;=\;f\left(\sum_{i}w_{i}x_{i}+b\right)$$

here, *w_i_* represents the weights, *x_i_* signifies the input, *b* denotes the bias term, and *f* is the activation function. The NN iteratively adjusts weights through forward and backward propagation to minimize the difference between predicted and actual values, continuing until a suitable convergence criterion is met. This iterative process refines the network's ability to perform complex recognition tasks.

Facing nearly ≈10^8^ types of HEAs can be developed from ≈64 elements in the periodic table, Singh et al.^[^ ^139^ ^]^ tested five vanilla algorithms including KNN, SVM, DT, RF, and XGBoost (XGB) concerning HEA fabrication using melting and casting manufacturing methods to predict the phase transitions in HEAs. Vazquez et al.^[^ ^140^ ^]^ employed a deep neural network (DNN)‐based model to identify frequent phases within the elemental system comprising Mn, Ni, Fe, Al, Cr, Nb, and Co. Actually, each algorithm possesses distinct situational strengths and weaknesses. For instance, SVM are predominantly utilized for classification tasks. Similarly, decision trees are well‐suited for classification but may exhibit overfitting concerns when the tree structure becomes excessively complex. The implementation of a voting mechanism in RF mitigates the risks associated with overfitting. **Table** **1** comprehensively outlines the advantages, disadvantages, and application contexts of select algorithms within these four principal categories of ML methodologies.^[^ ^141^ ^]^

**Table 1:** Comparison of machine learning (ML) algorithms used: advantages, disadvantages, and applications.^[^ ^141^ ^]^

Category	Algorithm^a)^	Advantages	Disadvantages	Applications
Supervised learning	Logistic regression	Simple, interpretable	Suitable for linearly separable problems only	Classification
	Support vector machine (SVM)	Handles linear/nonlinear data, effective in high dimensions	Long training time, less suitable for large datasets	Classification, regression
	Decision trees (DT)	Interpretability, automatic feature selection	Prone to overfitting	Classification, regression
	Random forests (RF)	Reduces overfitting, parameter tuning often unnecessary	Less interpretable	Classification, regression
	K‐nearest neighbors (KNN)	Simple	High storage and computational cost	Classification, regression
	Gaussian process	Highly flexible, no need to specify model form	Computationally intensive	Regression, classification
	Neural networks (NN)	Handles complex problems, many parameters	Requires large data, long training times	Classification, regression, feature extraction
Unsupervised learning	K‐means	Easy to understand, fast	Need to specify number of clusters, sensitive to initial values	Clustering
	Hierarchical clustering	No need to preset number of clusters	Computationally intensive	Clustering
	Principal component analysis (PCA)	Dimensionality reduction, retains information	Might lose critical information	Data analysis, dimensionality reduction
	Autoencoders	Feature learning, anomaly detection	Training can be complex	Dimensionality reduction, anomaly detection
	Apriori	Straightforward	Might be inefficient on large datasets	Frequent itemset mining
	Eclat	Efficient, requires a single scan	May need significant memory	Frequent itemset mining
Reinforcement learning	Q‐learning	Model‐free, easy to implement	Possibly inefficient in large state/action spaces	Reinforcement learning
	Deep Q‐network (DQN)	Handles high‐dimensional and continuous spaces	Requires significant computational resources	Reinforcement learning
	Policy gradient methods	Directly optimizes policy	May suffer from high variance	Reinforcement learning
Deep learning	Deep feedforward networks	Large model capacity	Needs vast data	Classification, regression
	Convolutional neural network (CNN)	Efficient for image processing	Primarily for image data	Image classification, object detection
	Recurrent neural network (RNN)	Handles sequence data	Training can be challenging	Sequence prediction, text generation
	Long short term memory (LSTM)	Addresses RNN's long‐term dependencies issue	Training might be slower	Sequence prediction, text generation
	Transformer	Parallel processing, handles long‐distance dependencies	Requires vast data	NLP, language modeling, machine translation

^a)^ Some algorithms can be used in different categories.

In the development of deep learning, several advanced models have significantly extended the field, including convolutional neural networks (CNNs), recurrent neural networks (RNNs), long short‐term memory networks (LSTMs), and transformer models. Each model has a unique structure and purpose, effectively handling various complex data and challenges.

CNNs are specifically designed to process data with a clear grid‐like structure, such as images. CNNs use convolutional layers to locally connect and share weights, significantly reducing the model's parameter count and enhancing the efficiency and accuracy of image processing. For instance, in image recognition and video analysis, CNNs can identify and classify objects within images.

RNNs excel in processing sequential data, such as text or time series data. Through their recurrent network structure, RNNs retain information from a previous state and use it to influence the output of the current state. This feature makes RNNs exceptionally effective in speech recognition, natural language processing, and other sequence prediction tasks.

LSTMs are a special form of RNNs that address the issue of vanishing or exploding gradients in traditional RNNs when processing long sequence data. LSTMs introduce three gates (input, forget, and output gates) to regulate the inflow, retention, and outflow of information, thereby maintaining long‐term dependencies in tasks, such as machine translation and text generation.

Transformer models, with their unique attention mechanism, can process data independently of its sequential nature. This makes Transformers more efficient in handling large volumes of data and exhibits outstanding performance in natural language processing tasks, such as text translation and sentiment analysis.

A team from institutions in the USA and China introduced a new machine learning‐based protocol—deep potential molecular dynamics (DPMD).^[^ ^142^ ^]^ DPMD can simulate systems with over 100 million atoms and complete trajectories longer than 1 ns within a single day, a feat unimaginable with traditional molecular dynamics methods. These advanced deep learning models and the application of DPMD demonstrate the immense potential of machine learning in handling large‐scale and complex data, opening new possibilities for scientific research and industrial applications. These advanced deep learning models and the application of DPMD demonstrate the immense potential of machine learning in handling large‐scale and complex data, opening new possibilities for scientific research and industrial applications.

### 3.5 Limitations of Current Data‐Driven Materials Discovery Techniques and Potential Solutions

**Figure 9:**
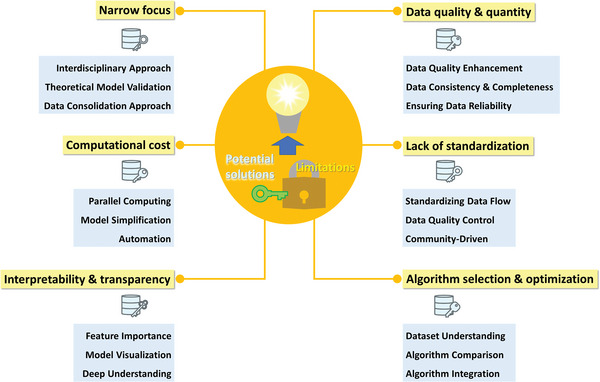
Limitations of current data‐driven materials discovery techniques and potential solutions.

Data‐driven methods analyze large datasets to identify patterns and correlations for making predictions, generating new hypotheses, or uncovering hidden mechanisms. This approach quickly processes vast amounts of data, leading to faster results and more accurate predictions with less reliance on expert knowledge. However, as shown in **Figure** **9**, there are still some limitations that need addressing to fully realize their potential. This section discusses these limitations and suggests potential solutions.

#### 3.5.1 Data Quality and Quantity

The accuracy and reliability of predictive models depend heavily on the quantity and quality of the data used to train them. The influence of data quality contains three fundamental domains: data accuracy, completeness, and consistency. Data accuracy stands as the linchpin of dataset quality, with inaccuracies potentially yielding erroneous conclusions. The correlation degree of curves fitted by scatter plots should be considered in the data collection process. Data completeness is closely linked to whether the dataset encompasses all requisite information. This entails verifying the inclusion of comprehensive experimental conditions and ensuring a balanced data distribution of specific variables within the dataset. Data consistency pertains to the uniformity of data within each class and across the dataset as a whole, including aspects such as unit conformity and format consistency. Inconsistencies in data pose a significant risk, as they can lead to excessive deviations in model fitting. The quantity of data also wields a profound influence, impacting both model complexity and data reliability. Data reliability holds significance in its ability to mitigate the risks associated with noisy and anomalous data, reinforced by multiple data points. Increasing data volume consequently reduces the influence of distribution variability on accuracy, contributing to a more robust model performance.

Solution: Data cleaning and preprocessing techniques are used to improve data quality. Additionally, material databases, experimental measurements, and simulations can also be used to increase the amount and diversity of available data. Following the data collection phase, the identification and management of outliers should be carefully handled. In order to maintain the consistency of data, the unified unit can effectively reduce the error caused by unit differences after data collection. For ensuring data completeness, the generation of distribution graphs for specific variables aids in assessing the dataset's comprehensiveness. A greater degree of uniformity within the distribution signifies enhanced data completeness.

#### 3.5.2 Lack of Standardization

Data collected from different sources is difficult to reach a uniform standard. Consequently, the issue of data consistency across multiple sources poses a substantial hurdle. For identical types of experiments, different laboratories may employ distinct methodologies or employ dissimilar instruments for characterization, resulting in disparities within ostensibly identical data categories. These inconsistencies hinder meaningful data comparisons. Moreover, the problem of incomplete data is prevalent among some sources, and data format inconsistencies further complicate data integration and cross‐source comparisons. Additionally, differing data representations, such as tables, images, and text, require manual processing or software‐driven transformation into a standardized format. Consequently, the development of robust data harmonization and quality assurance strategies remains imperative to mitigate these challenges effectively.

Solution: Researchers may establish standardized protocols and guidelines for data collection, storage, and sharing in materials research. Implementing data quality control measures, such as rigorous data cleaning and validation processes, can help identify and rectify any inconsistencies or inaccuracies in the collected data. In addition, promoting open and transparent data sharing practices can encourage researchers to openly publish their data and methodologies. This can enable the wider scientific community to assess, validate, and verify the data, thus ensuring its quality and reliability. This concerted effort not only empowers researchers to harness diverse datasets effectively but also serves as a catalyst for advancements and innovations in the field of materials science.

#### 3.5.3 Algorithm Selection and Optimization

A diverse array of ML algorithms exists, each offering unique advantages and constraints, making it essential to choose the appropriate algorithm for the task at hand. Related problems encompass a spectrum, including classification, clustering, and regression. Classification involves the segmentation of data into distinct groups based on provided labels. Clustering, conversely, entails the unsupervised categorization of data into groups without relying on predetermined labels. Regression concerns itself with predicting output values based on input parameters. The choice of algorithm hinges on the nature of the specific problem at hand. The volume of data also plays a pivotal role in algorithm selection. Certain algorithms, like GB, tend to perform optimally when deployed on extensive datasets, in contrast, algorithms such as KNN are adept at handling high‐dimensional data, executing classification or regression tasks based on sample distances. Furthermore, the selection process should consider the trade‐off between accuracy and computational complexity.

Solution: It is imperative for the investigator to possess an exhaustive comprehension of the available dataset. To this end, the initial stage necessitates the identification of fundamental elements, namely, the problem classification, the dataset's intrinsic characteristics, and the intended output parameters. A comprehensive comparative evaluation of diverse algorithms should be conducted to assess their efficacy with respect to specific dataset under consideration. In addition, hyperparameters profoundly influence the performance of most algorithms. Employing hyperparameter optimization techniques becomes instrumental in fine‐tuning the algorithms’ behavior. By discerning the optimal hyperparameter configurations for each algorithm, researchers can maximize their efficacy.

#### 3.5.4 Narrow Focus on Certain Materials

Data‐driven approaches could suffer from the possible bias of being limited to the particular material system under investigation. This limitation could lead to underdeveloped models and limited generalization to unknown materials. Excessive fixation by researchers on specific materials carries the inherent risk of overlooking alternative materials with untapped potential, thereby constraining the scope of material innovation. It is essential to recognize that research outcomes pertaining to a particular material system may not readily translate to diverse materials, which is attributed to the inherent diversity in material properties and behaviors.

Solution: Researchers must focus on an interdisciplinary approach that includes theoretical insights, experimental data, and the application of the knowledge gained from all material systems. This approach can guarantee the optimization of the models’ reliability and applicability. Primarily, theoretical insights possess an inherent capacity for broader applicability across different material systems, which can help researchers to formulate models characterized by a higher degree of generality. Then the empirical validation through experimental data serves to delineate the scope of models’ applicability. An integrated approach, involving the amalgamation of data originating from disparate material systems into a unified framework through standardized practices, represents an avenue for achieving a more comprehensive understanding of material behavior.

#### 3.5.5 Interpretability and Transparency

The lack of interpretability and transparency associated with complex algorithms can lead to skepticism regarding their reliability and makes it difficult for researchers to gain insights into the underlying relationships between materials properties. In certain computational tasks, the decision‐making process of the model unfolds through a multitude of intricate layers and successive steps. This complexity presents a formidable challenge in terms of elucidating and comprehending the underlying rationale for each decision made. The inherent opacity and absence of transparency within such models pose significant impediments. The deficiency in providing coherent explanations and insights detracts from the model's credibility, impedes its acceptance, and introduces obfuscation into the interpretation of experimental outcomes and predictions engendered by the model.

Solution: Researchers can adopt techniques, such as feature importance ranking, that offer an interpretation of the models’ predictions. Additionally, transparent models that prioritize interpretability, such as decision trees and linear regression models, can help researchers gain insights into the underlying mechanisms and relationships. The utilization of model visualization techniques represents a valuable strategy for elucidating the intricacies of the decision‐making process inherent to the model. In certain instances, an equilibrium between interpretability and accuracy can be attained by judiciously curtailing the model's complexity. In addition, it is important to cultivate a profound understanding of the model's architecture and the physicochemical intension of the input features and output targets from the view of materials science.

#### 3.5.6 Computational Cost

Modeling and design of materials using data‐driven techniques can be computationally intensive, requiring significant computational resources. Computational resources include hardware resources, such as central processing unit (CPU), graphics processing unit (GPU), memory, hard disk, etc. Different data‐driven methods have different demands on hardware resources. Meanwhile, the storage and use of large‐scale datasets will have a greater demand for hard disks. Complex models and large‐scale datasets can take a lot of time which further increases the computational cost. In addition, in order to evaluate the best model, the model is usually trained and evaluated multiple times, as well as fine‐tuning the hyperparameters for optimization, which can lead to additional computational costs. These above factors can limit the speed of model training, evaluation, and optimization.

Solution: Computational efficiency in material discovery can be improved by developing more optimized and parallelized algorithms. Additionally, leveraging high‐performance computing platforms and technologies can help distribute the computational workload and reduce the processing time required. Streamline the data collection and preprocessing stages by minimizing redundant or noisy data and utilizing dimensionality reduction techniques can also tackle the computational cost challenge. As for model design, one effective strategy entails streamlining complexity, notably by reducing the number of layers within NN and optimizing the number of neurons. This approach not only truncates training durations but also preserves performance metrics. At last, the integration of an integration algorithm, particularly the amalgamation of a genetic algorithm into the core model, presents an efficacious approach for hyperparameter optimization, which facilitates an exhaustive search of hyperparameter configurations, executed at a relatively expedited pace.

In summary, technical challenges such as data quality, standardization, algorithm selection, narrow focus, interpretability and computational cost can pose significant hurdles to the effectiveness of data‐driven approaches. However, researchers can still address these challenges through multiple solutions, which include data cleaning, standardized protocols, comparative algorithm performance analysis, interdisciplinary approaches, data processing streamline, among others.

## 4 Typical Applications of Data‐Driven Investigations for Functional Coatings

Efficient data‐driven approaches have gradually permeated modern new materials development strategies. In the development of functional coatings, many researchers are beginning to try to use ML to develop new materials. The follow sections will demonstrate the potential of data‐driven approaches in functional coatings development through several case studies. They highlight the advantages of using data‐driven approaches, especially ML, to accelerate the discovery of new coatings with improved performance.

### 4.1 Anticorrosion Coatings

Corrosion is a primary failure form for most materials. Since it is inevitable, mitigating corrosion has become a key focus of research to overcome this challenge. Functional coatings serve as protective films, which effectively extend the service life of the materials. Corrosion inhibitors are considered to be one of the most effective anticorrosion functional coatings, whose main functional component is organic molecules. In general, corrosion inhibitors reduce the corrosion by absorbing water and oxygen on the surface of the materials. Therefore, the interfacial adsorption, hydrophilic, and dense properties of corrosion inhibitor molecules directly affect the protection ability of anticorrosion coatings. These characteristics, including chemical structure, charge distribution, film thickness, and roughness, are used as molecular descriptors in data‐driven models to establish linear relationships with material property/activity, known as quantitative structure–property/activity relationships (QSPR/QSAR), thereby predicting or designing effective corrosion inhibitor molecules.^[^ ^143^ ^]^

**Figure 10:**
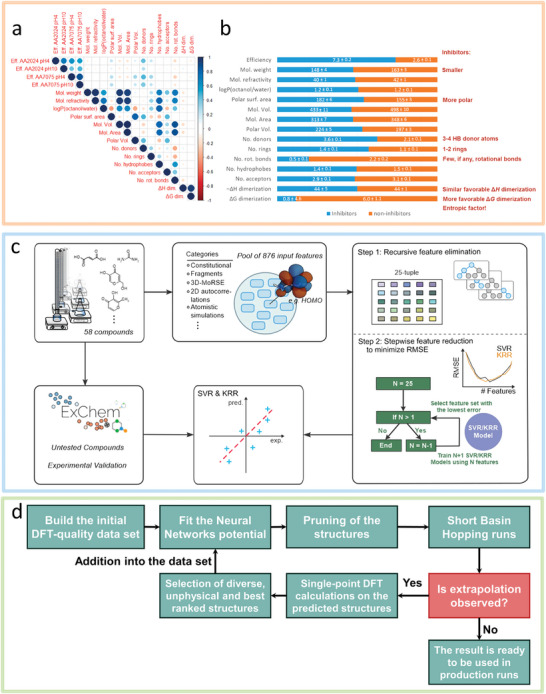
a) Linear correlation plot from the regression analysis of the inhibition efficiencies and descriptors modeled. Blue represents a positive linear correlation, while red represents a negative linear correlation. Larger circles mean a higher absolute linear correlation value. b) Percentage stack plot of the average properties of inhibitors and noninhibitors with the respective standard error (left) and main characteristics of corrosion inhibitors in comparison with noninhibitors (right). Reproduced under the terms of the CC‐BY license.^[^ ^144^ ^]^ Copyright 2020, the Authors, published by American Chemical Society. c) A training database of 58 small organic molecules and their corrosion responses on AZ91. Reproduced under the terms of the CC‐BY license.^[^ ^146^ ^]^ Copyright 2023, the Authors, published by Springer Nature. d) Training protocol flow chart. Reproduced with permission.^[^ ^148^ ^]^ Copyright 2021, Elsevier.

Considering plentiful candidate organic molecules with potentially useful properties, the traditional trial‐and‐error method is not only time‐consuming and labor‐intensive, but also may cause resource waste and environmental pollution. Fortunately, data‐driven approaches facilitate rapid exploration of available corrosion inhibitors on the ground of QSPR/QSAR. Among these, ML is the most widely used method. First, ML methods are able to quickly screen functional organic molecules (**Figure** **10**). Galvão et al.^[^ ^144^ ^]^ used different algorithms to classify 102 organic compounds to distinguish whether they are effective corrosion inhibitors for Al alloys in aviation applications. A large number of descriptors, such as molecular weight, octanol/water partition coefficient, polar surface area, dimerization enthalpy, and Gibbs energy, etc., are used. The linear correlation between these descriptors modeled and inhibition efficiencies can be reflected in Figure 10a, which indicated that identifying molecular structures that can be used as corrosion inhibitors is a highly nonlinear problem. Figure 10b presents the corrosion inhibitor characteristics obtained through training. That is, molecules with one or two aromatic rings, few or no rotational bonds, strong polarity, and more favorable dimerization Gibbs energy are beneficial for corrosion inhibition. Second, ML is also used for designing corrosion inhibitors. For instance, based on structural similarity and kernel ridge regression (KRR), ML models predict the ability of small organic compounds to modulate the corrosion of commercially pure Mg.^[^ ^145^ ^]^ Similarly, Li et al.^[^ ^146^ ^]^ utilized support vector regression (SVR) and KRR to acquire the corrosion response of 58 small organic molecules on Mg alloy and encoded their molecular information using the molecular descriptors calculated by their geometry and DFT. Figure 10c shows the training database. First, a pool of 2876 different molecular descriptors were generated as input features of the model, and the features were selected. The most relevant descriptors are then used to train ML models to predict the behavior of untested chemicals. The results were finally tested experimentally.

In addition, high‐throughput experiments are also one of the ways to rapidly develop corrosion inhibitors. The coupling of ML and high throughput experiment can not only improve the sieving efficiency of corrosion inhibitor molecules and enabling quantitative prediction of material properties, but also accelerate the validation of predictions. In search of effective alternatives to toxic chromate corrosion inhibitors, Winkler et al.^[^ ^147^ ^]^ combined high‐throughput corrosion screening techniques and modern ML methods to obtain the molecular structure‐corrosion inhibition relationship for a large number of corrosion inhibitors applied to aerospace Al alloys. Deservedly, metal coatings are another commonly used anticorrosion coating. Álvarez‐Zapatero team presented a NN potential capable of predicting the structure of pure and hybrid nanoparticles.^[^ ^148^ ^]^ The specific training protocol is shown in Figure 10d. In comparison with the values provided by DFT used in the training process, the NN potential able to predict structures with an error in energy and forces of the order of chemical accuracy. Specifically, ZnMg nanohalloys with up to 52 atoms were tested and analyzed for structural properties, chemical order, stability and electronic information, to determine whether they could be used as effective corrosion‐resistant coating compositions.

### 4.2 Temperature‐Resistant Coatings

**Figure 11:**
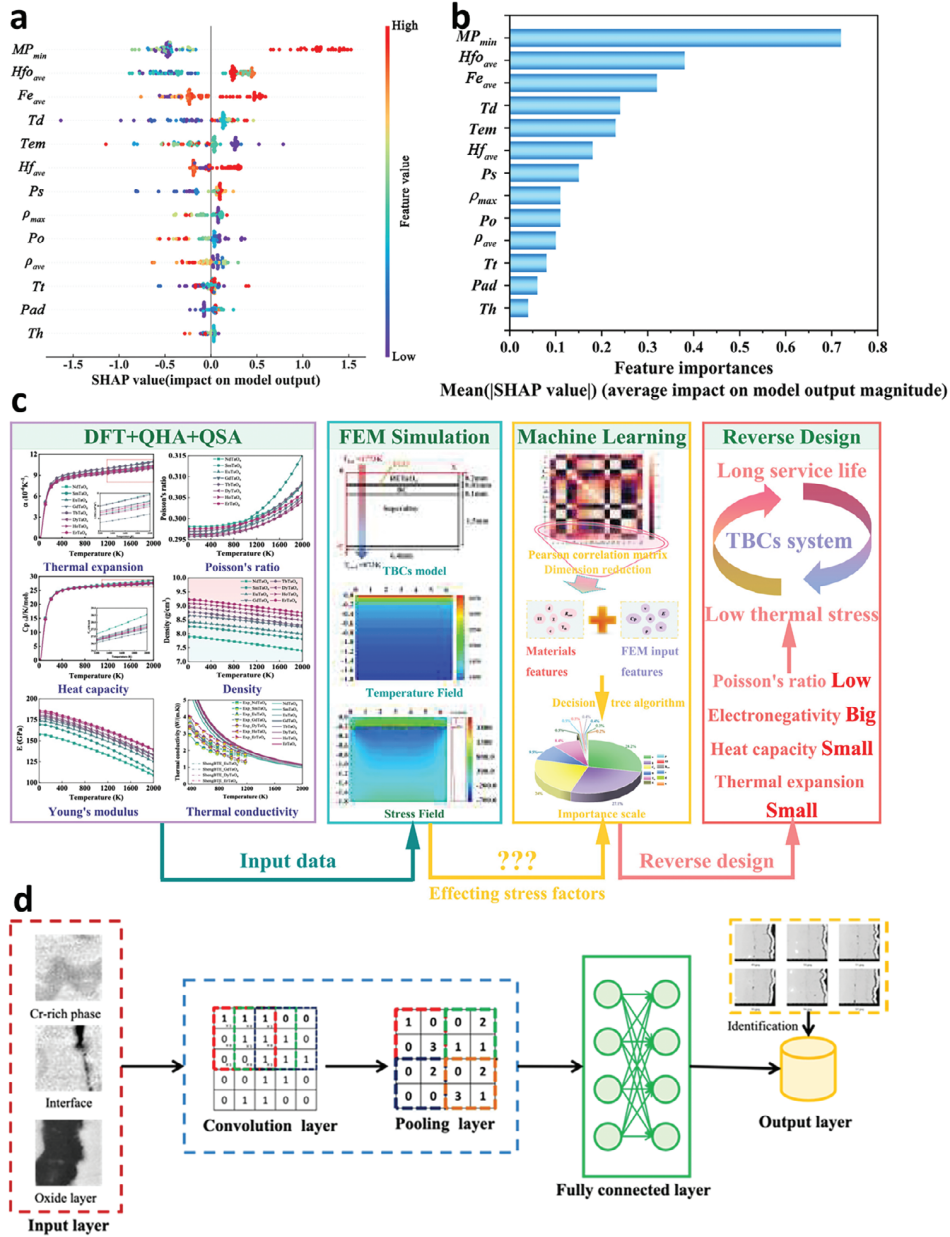
a) SHAP values and b) |SHAP| values affecting oxidation and ablation to clarify the failure mechanisms of ultrahigh‐temperature ceramic coatings. Reproduced with permission.^[^ ^149^ ^]^ Copyright 2022, Elsevier. c) A multiscale evaluation method coupled with DFT calculations, FEM, and ML, can be used for reverse design for thermal barrier coating. Reproduced with permission.^[^ ^150^ ^]^ Copyright 2024, Elsevier. d) The architecture of the convolutional neural network for recognizing the characteristic phases of the NiCrAlY coating after oxidation. Reproduced under the terms of the CC‐BY license.^[^ ^151^ ^]^ Copyright 2022, the Authors, published by Springer Nature.

High‐temperature protection coatings are design to provide thermal insulation or withstand extreme heat, which are used to prevent surface oxidation and performance degradation of high‐temperature equipment in various fields, such as aerospace, nuclear energy, and manufacturing. The service performance improvement is always accompanied by an increase in working temperature. However, typical heat‐resistant materials, such as ceramics or refractory metals, may suffer from phase or structure transformations at high temperatures, leading to a significant accident caused by material damage. The data‐driven design of temperature‐resistant coatings based on the data ocean has been developing for a long time (**Figure** **11**). Hao et al.^[^ ^149^ ^]^ adopted ML based on experimental datasets to build a model for predicting the oxidation and ablation resistance of ultra‐high‐temperature ceramic (UHTC) coatings. 13 factors were considered, and SHapley Additive exPlanations (SHAP) was used to derive each factor's priority in affecting the coatings' performance (Figure 11a,b). The mass ablation rates of UHTC coatings increase with melting point (Mp_min_), the enthalpy change of oxidation reaction (Hfo_ave_), standard enthalpy of formation (Hf_ave_), the temperature of the sample surface (Tem), the powder's particle size (P_S_), and formation energy (Fe_ave_), but negatively correlate with the density (*ρ*_max_ and *ρ*_ave_) and the porosity of the coating (P_O_) (Figure 11a).The factor importance (|SHAP|) shows that Mp_min_ is the most critical factor for predicting mass ablation because of the liquid phase without protection (Figure 11b).

Thermal barrier coatings (TBCs) are important in gas turbine engines, power‐generation turbines, and rocket engines. However, the effect of thermal stress on TBCs stability and durability remains difficult to assess, which has puzzled scientists until a new study presented recently by Zhong et al.^[^ ^150^ ^]^ They developed a cross‐scale model that combines DFT calculations, thermodynamic calculations, FEM, and ML to evaluate the thermal stress of TBCs (Figure 11c). Taking rare earth tantalate as an example, the thermal expansion coefficient, heat capacity, thermal conductivity, Young's modulus, Poisson's ratio, density, and other properties were obtained by DFT calculation. Then coupled with FEM, the temperature field and stress field distribution were quantified and visualized. Furthermore, the decision tree with thermal stress as the target property was established. The important features affecting thermal stress were ranked to found the features related to low thermal stress, including small Poisson's ratio, strong electronegativity, small heat capacity, and thermal expansion coefficient. Finally, the rationality and reliability of the low‐stress mapping characteristics obtained by ML are verified by using YTaO_4_ and double rare earth tantalate (Ho_0.5_Er_0.5_)TaO_4_ which are not available in the data set.

As material genetic engineering develops by leaps and bounds, the prediction of material properties can be achieved based on visual analysis technology. As one of the traditional protective methods, the protective coating has made it difficult to achieve accurate prediction of coating life due to its service process covering many factors and complex failure factors. Liu et al.^[^ ^151^ ^]^ collected a large number of topography images during the service of the coating and through visual analysis technology to establish image preprocessing screening and standardization. A multilayer convolutional neural network (8–10 layers) was established to automatically identify the characteristic phase of the signal for distinguishing the coating, coating/substrate interface, and corrosion product layer (Figure 11d). Based on the measured results of the coating performance, the neural network was used to establish an effective network correlation between image quantitative and performance quantitative. The image simulation and reconstruction simulation network of coating changes under different service time were established, to realize the simulation of coating service performance image state and life prediction.

### 4.3 Wear‐Resistant Coatings

**Figure 12:**
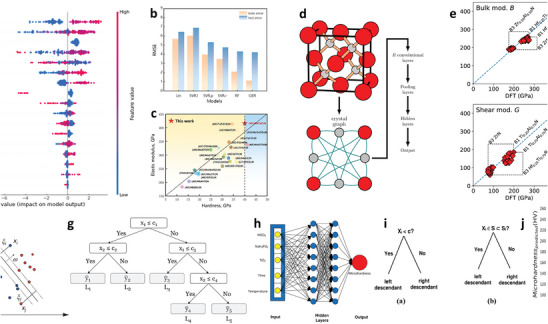
a) Global interpretation using SHAP. b) Train error and test error of different machine learning models. c) The optimal hardness and elastic modulus of screened composition by ML and high‐throughput experiment compared with other works. Reproduced with permission.^[^ ^152^ ^]^ Copyright 2022, Elsevier. d) The sketch map of CGCNN transforms the crystal structure into a crystal graph and then outputs the result as a variable input through a series of processes. e) The disordered compound's predicted and calculated elastic constants are highly consistent. Reproduced under the terms of the CC‐BY license.^[^ ^74^ ^]^ Copyright 2022, the Authors, published by Springer Nature. The algorithm diagram of f) SVM, g) ANN, h) RF, and i) ET. j) Predicting the microhardness of Ni‐P‐TiO_2_ composite coating by ET. Reproduced with permission.^[^ ^154^ ^]^ Copyright 2023, Elsevier.

Among a variety of properties of functional coatings, wear resistance has been regarded as a critical one, especially for the surface protection of moving components of machineries. Based on data‐driven approaches, the role of key microstructural, property, and environmental parameters on the tribological behaviors can be isolated to guide the design of wear‐resistant coatings. Xu et al.^[^ ^152^ ^]^ screened high‐entropy coating combining the ML and high‐throughput magnetron sputtering experiment (**Figure** **12**a–c). The SHAP suggested that high values of bias voltage, N, Te, and Ta, and low values of Ni and Cr were the key factors of the coatings hardness by training the data of 167 pieces of literature (Figure 12a). Six types of ML algorithms were performed, including linear regression (Lin), linear kernel support vector regression (SVR.l), polynomial kernel support vector regression (SVR.p), radial kernel support vector regression (SVR.r), RF, and gradient boosting regression (GBR) models, to establish the relationship between seventeen variables and hardness. The train error and test error of different ML algorithms showed RF and GBR performed well (Figure 12b). But GBR had an overfitting phenomenon. RF was selected to guide the preparation parameters of coatings. The high‐throughput magnetron sputtering experiments found the hardness of (AlCrNbTaTi)N coating based on the prediction of RF reached 40.1 GPa, which proved the prediction model has great potential in the development of super‐hard high‐entropy ceramic coatings (Figure 12c). Their team further screened a new composition coating of (AlCrTiMoTa)N with a hardness of 33.4 GPa and modulus of 287.7 GPa through ML and multiobjective optimization from the database of high‐entropy ceramic coating.^[^ ^153^ ^]^ ML can also establish the relationship between 3D structure and performance. Levämäki et al.^[^ ^74^ ^]^ applied a method that combined DFT calculation and ML to predict elastic properties of hard‐coating alloys, such as Hf_1−_ *_x_* Al*_x_* N and Ti_1−_ *_x_* Al*_x_* N (*x* = 0, 0.25, 0.5, 0.75, 1). They used the database of elastic properties for order compounds in the material project to train the crystal graph convolutional neural network (CGCNN) (Figure 12d). The DFT calculation was performed to calculate the elastic constants of disordered compounds, but was rarely used due to the high cost. Then, the transfer learning (TL) approach established a relationship between the abundance of data for ordered compounds and more minor data for disordered compounds. The predicted bulk (B), shear (G), Young's (E) modulus, and Poisson's ratio *v* of the disorder compound are highly consistent with the calculated value (Figure 12e). Despite using a small amount of data from disordered compounds, it is still possible to accurately predict the data of unknown disordered compounds from the data of large ordered compounds and a few disordered compounds. This method might accelerate the discovery of new hard‐alloy coatings with disordered crystal structures. The influence of different treatment methods on the hardness of the coating can be predicted by ML. Shozib et al.^[^ ^154^ ^]^ applied four ML algorithms, including SVM, ANN, RF, and Extra Trees (ET), to predict the microhardness of the Ni‐P‐TiO_2_ coating (Figure 12f–i) obtained from different compositions and operation conditions of electroless plating bath solutions. The ET was considered a learning model that can minimize the experimental complexities, time, and expense in the manufacture of parts with good surface properties by predicting microhardness (Figure 12j). This method provides a new way to establish a strong relationship between surface modification and coating performance without experimental cost.

### 4.4 Antifouling Coatings

**Figure 13:**
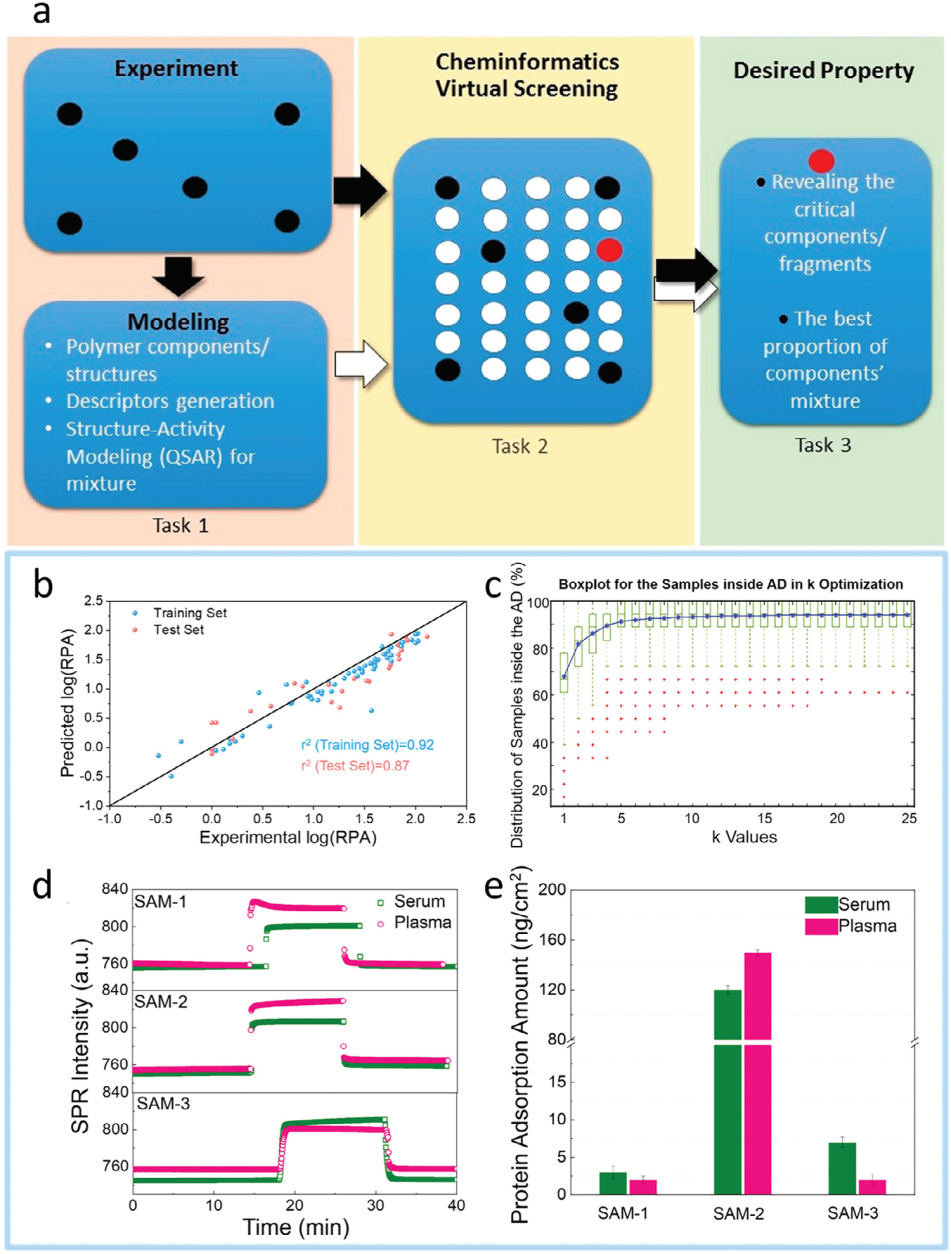
a) Schematic diagram of polymer coating modeling and design. Reproduced with permission.^[^ ^156^ ^]^ Copyright 2017, American Chemical Society. Validation of the predictivity and reliability of the ANN model: b) Plot of the predicted versus experimental log (RPA) for both training and test sets. c) Application domain analysis of 26 testing samples using the KNN approach. Experimental validation of the protein resistance property of the three designed SAMs: d) Protein adsorption sensing data of the three SAMs in undiluted human blood plasma. e) The final protein adsorption amount of the three SAMs. Reproduced with permission.^[^ ^158^ ^]^ Copyright 2021, American Chemical Society.

Materials can effectively extend their service life by preventing the surface from being attached to biological, inorganic, and organic matter. Early antifouling strategies are embedding metal and/or organometallic fungicides into the polymer matrix of the paint.^[^ ^155^ ^]^ The toxicity and biological harmfulness of fungicides seriously destroy the environment. Therefore, the modern antifouling policy aims at screening nontoxic and efficient antifouling polymers. The rational design of highly antifouling coatings is essential for fundamental research and applications (**Figure** **13**). The previously mentioned QSAR can be also used to design antifouling coatings. Rasulev et al.^[^ ^156^ ^]^ synthesized a set of 27 nontoxic, amphiphilic polysiloxane‐based polymer coatings by a combinatorial, high‐throughput approach. Schematic diagram of the overall steps is shown in Figure 13a. They proposed the variable selection GA and multiple linear regression analysis (MLRA) methods to develop QSAR model to predict fouling‐release activity of relevant marine fouling organisms, including bacteria, microalgae, and adult barnacles. It was found that the polarizability indices, size, mass, and van der Waals volume could significantly affect the antifouling performance of the coatings. In addition to the marine industry, biological antifouling in the medical sector is also crucial. The protein adsorbed on the surface of nanomaterials is an important medium at the bio‐nanomaterials interface. Therefore, antiprotein surface coatings can prevent biological contamination. Le et al.^[^ ^157^ ^]^ used ML methods to extract quantitative relationships between surface chemistry of materials and protein adsorption properties to reliably predict the degree of protein adsorption on the surface of materials, aiming to design effective antifouling coatings. Liu et al.^[^ ^158^ ^]^ developed a data‐driven ML model, combing factor analysis of functional group (FAFG), Pearson analysis, RF, and ANN algorithms as well as Bayesian statistics, to evaluate the antifouling performance of any given or designed self‐assembled monolayers. The key molecular descriptors and functional groups for the rational design of antifouling materials were identified. The ANN model was trained and verified by constructing datasets, as shown in Figure 13b,c. Through modeling and prediction, three kinds of antifouling molecules were synthesized and made into self‐assembled monolayers (SAMs). The experimental results showed that all three kinds of SAMs could adsorb proteins in undiluted fresh plasma. However, after rinsing with phosphate buffer solution, the proteins all fell off (Figure 13d), and the final adsorption amount was shown in Figure 13e, which indicated that SAMs had weak adsorption capacity for proteins, that is, protein resistance. The difference between SAM‐1 and SAM‐3 is that the ether group in SAM‐1 and the amide group in SAM‐3 contribute differently to their respective protein resistance, which is consistent with model predictions.

### 4.5 Self‐Healing Coatings

**Figure 14:**
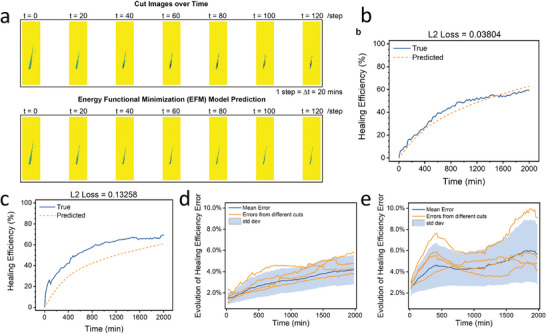
a) Key results of the shape of the cut at different healing times from the EFM model‐top row: input cut image data; bottom row: predicted cuts with ML model. The healing efficiency based on a training b) and test c) dataset. The blue line represents the true data and the orange dotted line represents the predicted data. Evolution of healing efficiency error based on training d) and test e) dataset. Orange lines represent the absolute errors between the true and predicted healing efficiency over time. The solid blue line is the average error, and the shadow indicates the standard deviation. Reproduced with permission.^[^ ^165^ ^]^ Copyright 2022, American Chemical Society.

Functional coatings are used to enhance the sustainability of the materials. However, during harsh conditions, whether they are anticorrosion coatings, wear‐resistant coatings, or antifouling coatings, all of them will be subjected to environmental or internal stress resulting in defects. In nature, biological organisms possess repair mechanisms to prevent loss of function, which involves processes of a series of physical/chemical reactions. Similarly, functional coatings on the surface of service materials can be sustainably applied as long as they are allowed to spontaneously heal and repair defects. Self‐healing coatings are divided into external self‐repair and intrinsic self‐repair according to repair types.^[^ ^159^ ^]^ External self‐healing coatings are pre‐embedded with additional components, such as microcapsules,^[^ ^160^ ^]^ carbon nanotubes,^[^ ^161^ ^]^ fibers,^[^ ^162^ ^]^ or nanoparticles containing,^[^ ^163^ ^]^ to realize self‐healing function. Under the action of external stimuli (force, pH value, temperature, etc.), the repair agents are released in the damaged region, so as to achieve self‐healing. External self‐healing methods are considered to have universal applicability for most coatings. The intrinsic self‐healing function is realized by the coating material itself containing special chemical bonds or other physical and chemical properties such as reversible covalent bonds, noncovalent bonds, molecular diffusion, etc. The latter eliminates complex steps such as pre‐repair agent embedding technology, and has little impact on substrate properties. The molecular structure design of coating substrate materials is a significant challenge before data‐driven methods are widely applied. The multichannel preparation and high‐throughput screening methods shorten the experimental time by nearly ten times. Manabe et al.^[^ ^164^ ^]^ built a ML database based on their experimental research to understand the effects of additives on repair times. By integrating ML with NN and gradient boosting decision tree (GBDT), cross‐validation was carried out with the experimental results. Using 14 descriptors as input parameters, they predicted that the horizontal/vertical swelling rate ratio, water absorption rate, and thickness were key factors in reducing self‐healing time. The model was then used to predict the Zn^2+^ doped coating, and it was found that the coating did not exhibit self‐healing ability, which is consistent with the experimental results. Therefore, it is possible to obtain an initial evaluation of coating properties by inputting the physical and chemical properties of polymers and additives as basic parameters through ML. However, the accuracy of the model needs to be optimized with more available data, otherwise there may be a high risk of overlearning. Fortunately, Anwar Ali et al.^[^ ^165^ ^]^ proposed a model to predict and understand the evolution of self‐healing properties of polymers using energy functional dynamics (SPEED) (**Figure** **14**). Using ML method, the energy function minimization (EFM) model describes the physical mechanisms of self‐healing in terms of potential and interfacial energies by extracting an effective underlying dynamical system from a time series of 2D cut images of self‐healing polymers of constant thickness. As shown in, the EFM model can predict the healing time of different incision shapes with high prediction accuracy (Figure 14a), and the prediction results of training data (Figure 14b) and test data (Figure 14c) show low healing efficiency errors (Figure 14d,e). The SPEED model, in combination with a static performance prediction model, enables the prediction of macroscopic material properties by training only a small set of experimental measurements evolution.

### 4.6 Electrically Correlated Coatings

#### 4.6.1 Electronic Coatings

**Figure 15:**
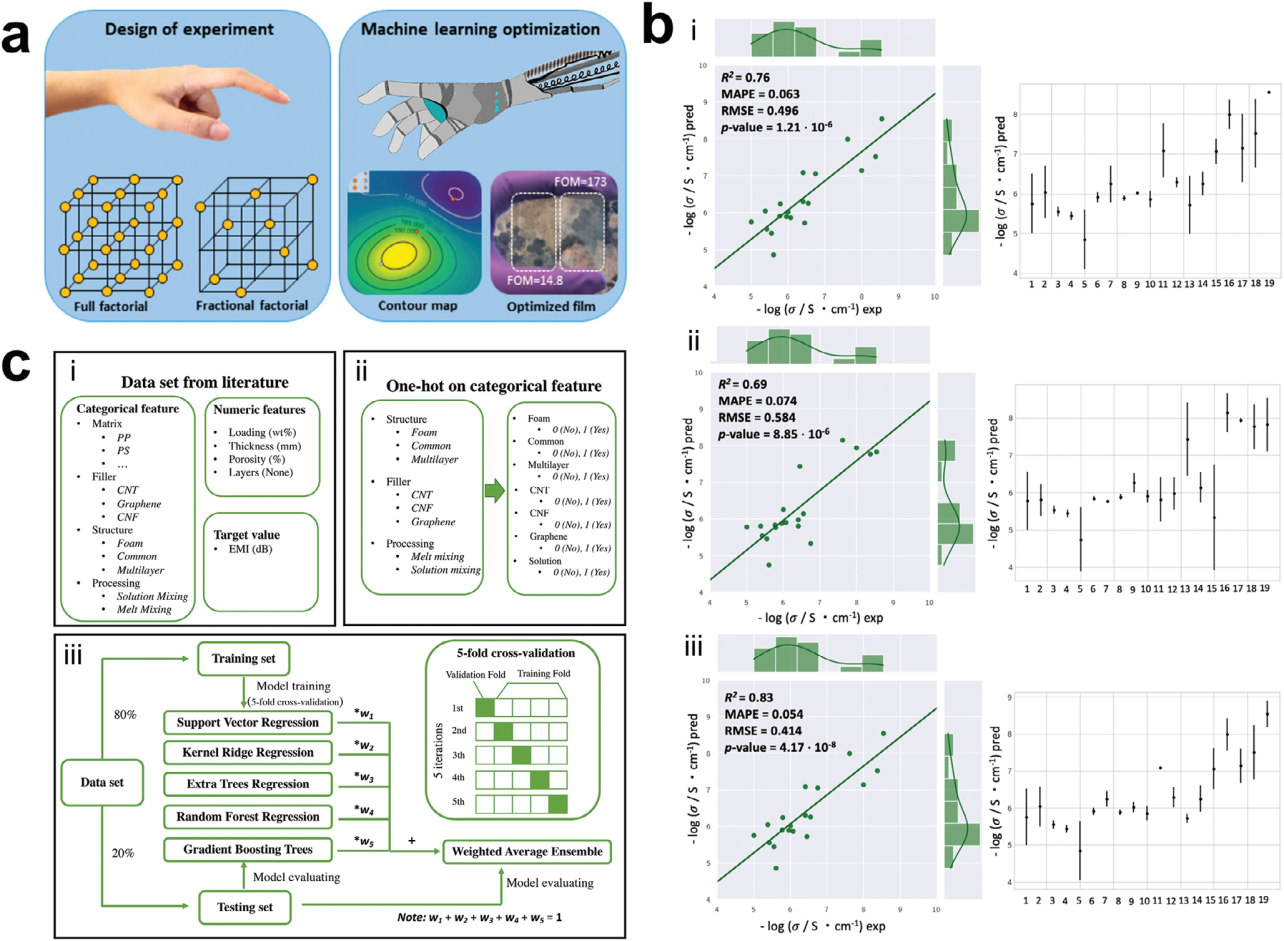
a) Aims of machine learning optimization. Reproduced with permission.^[^ ^166^ ^]^ Copyright 2019, American Chemical Society. b) Experimental versus predicted Li‐ion ‐log conductivity values for LSTM (i), PBP (ii) models and their averaged prediction values (iii). Reproduced under the terms of the CC‐BY license.^[^ ^170^ ^]^ Copyright 2023, the Authors, published by MDPI, Basel, Switzerland. c) The composition of data set (i); schematic diagram of one‐hot on categorical features (ii); schematic diagram of the model training process, including fivefold cross‐validation (iii). Reproduced with permission.^[^ ^172^ ^]^ Copyright 2019, Elsevier.

Electronic materials determine the pace of intelligent development, and the widespread adoption of electronic devices has promoted the research into electronic materials. Among them, the application of electronic coatings is particularly extensive, due to their high electrical conductivity, electromagnetic shielding, and antistatic properties. For refining the properties and improving the functionality of coatings, data‐driven approaches have been extensively utilized. Flexible electronic materials are a key component in the research of wearable electronic devices and also an important guarantee for the development of high‐tech and intelligentization. p‐TCMs serve as critical elements within optoelectronic devices such as solar cells, photodetectors, displays, and flexible sensors. Wei et al.^[^ ^166^ ^]^ demonstrated how ML algorithms, specifically SVR, could efficiently optimize the performance of p‐Type transparent conducting materials (p‐TCMs) by considering multiple deposition parameters (**Figure** **15**a). The approach involved calculating a figure of merit (FOM) based on coating conductivity and optical transmission, followed by two rounds of optimization to identify optimal areas in the parameter space. Wu et al.^[^ ^167^ ^]^ introduced a novel ionic conductive hydrogel skin material that exhibited excellent stretchability, strength, toughness, elasticity, transparency, self‐healing ability, and stable electrical cycling performance. By integrating a ML module, the hydrogel ionic skin showed tremendous potential in identifying complex human behaviors, such as writing in the air or on paper. The algorithm processed current signals generated from finger movements during writing, achieving high accuracy in predicting and recognizing letters, words, phrases, and short sentences. Certainly, electronic coatings also have extensive applications in electrochemical energy storage. Chen et al.^[^ ^168^ ^]^ constructed a ML model using 738 sets of data on the relative dielectric constant of polymers, to predict 11 000 potential polymer candidates. Subsequently, 10 sets of dielectric polymers were investigated. Zhu et al.^[^ ^169^ ^]^ utilized ML to fine‐tune the properties and arrangement of nanofillers, resulting in the creation of a composite material that exhibited high electric breakdown strength and high energy storage density. Kireeva et al.^[^ ^170^ ^]^ analyzed the existing experimental data of garnet thin coatings in solid‐state batteries, focusing on how ionic conductivity was affected by factors such as the lattice mismatch between the coating and the substrate, variations in elastic properties, and inconsistencies in thermal expansion characteristics. Additionally, the impact of the deposition temperature of the coating, the melting point, and the dielectric constant of the substrate on conductivity was investigated. Utilizing the findings from this analysis, a quantitative model for predicting the ionic conductivity of garnet thin coatings was established by using LSTM and Probabilistic Backpropagation (PBP) neural network, as shown in Figure 15b. Interestingly, insulating and separator materials play a vital role in energy storage devices, ensuring their reliable operation. Some electronic devices require insulation from external interference to function properly. Salah et al.^[^ ^171^ ^]^ utilized ML to predict the electromagnetic absorption performance of polycarbonate/carbon nanotube composite materials. By developing a new system, 15 different multilayer perception (MLP) networks were established, each specialized in predicting the absorption value of specific category samples. The research results demonstrated that the system performed exceptionally well in the database, with an average accuracy of 99.7997% and an overall average calculation time of 0.01 295 s. Additionally, the composite based on polycarbonate‐5 wt% carbon nanotube was found to be the ultimate absorber over the microwave range, in line with the requirements of Rozanov formalism. Shi et al.^[^ ^172^ ^]^ developed a fast predictive ML model for Carbon‐based fillers/polymer nanocomposites to shield electromagnetic interference. Relevant features are classified as categorized as categorical or numerical features, as shown in Figure 15c. It was found that the following key features: filler loading, thickness, porosity, and the number of layers, needed to be considered in the design of coatings, and the former should be paid more attention. Such multifunctional electronic coatings using data‐driven development have excellent performance, providing strong evidence for data‐driven development of more novel and better performance electronic coatings.

#### 4.6.2 Solar Cell Coatings

**Figure 16:**
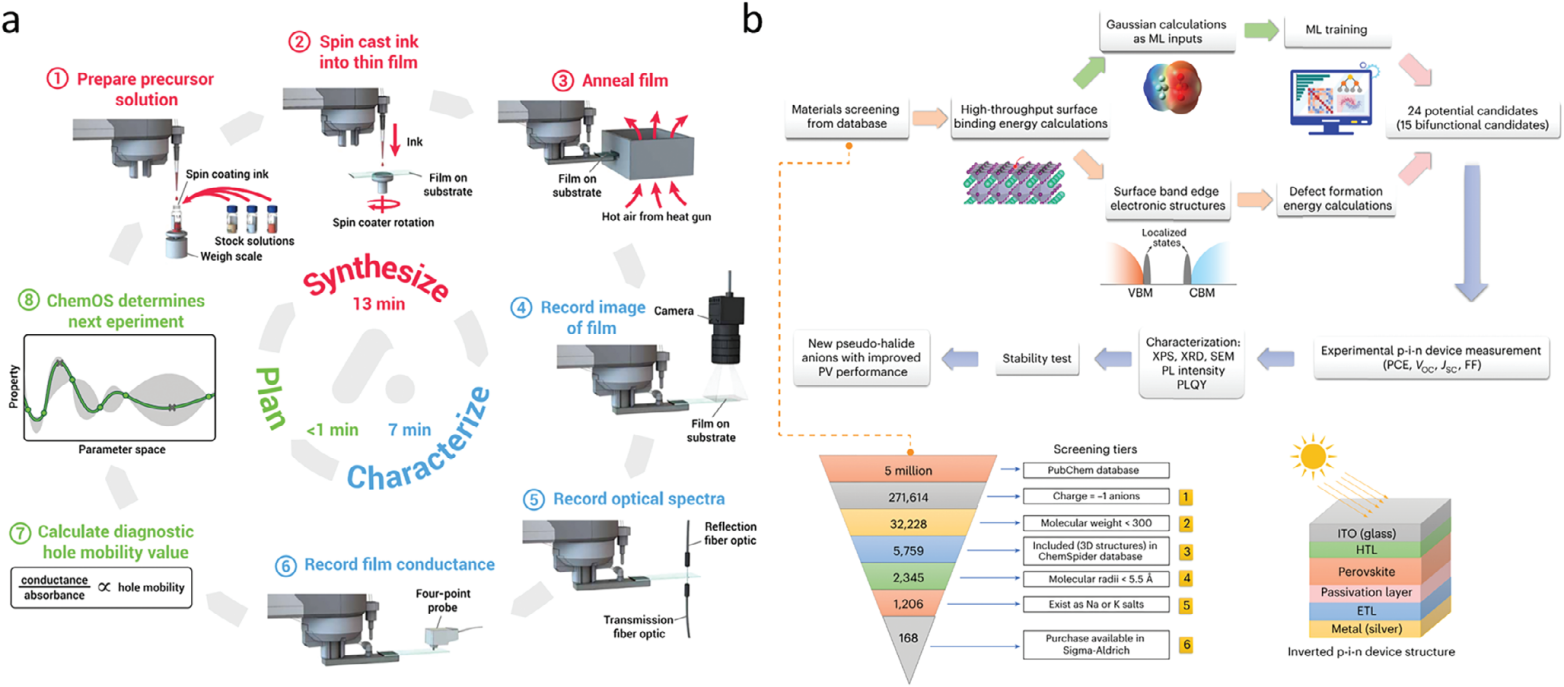
a) Schematic of Ada's automated workflow consisting of three main parts: Synthesize, Characterize, and Plan. Reproduced with permission.^[^ ^173^ ^]^ Copyright 2020, American Association for the Advancement of Science. b) Material screening process workflow with six screening tiers. VBM, valence band maximum; CBM, conduction band minimum. Reproduced with permission.^[^ ^174^ ^]^ Copyright 2023, Springer Nature.

Solar energy, as a kind of clean energy, has been widely concerned and applied in power generation industry. However, in order to improve the conversion efficiency of solar energy, solar cells are often designed to be large, resulting in expensive manufacturing. Therefore, with the aim of improving the service life, solar cell protective coatings play a key role. The coatings should not only be superhydrophobic, antifouling, and anticorrosion, but also ensure a good light transmittance to ensure the photoelectric conversion efficiency. MacLeod et al.^[^ ^173^ ^]^ established a self‐driving laboratory named “Ada” to automatically complete the sample preparation, detection, optimization, and screening for the organic hole and electron transport layer or optoelectronics application film. **Figure** **16**a shows the automated workflow of Ada. First, the precursor solutions in different ratios of films are mixed and dropped on the surface of the substrate, which are solidified into coatings under a heated airflow. Second, morphology, spectrum, and conductivity of the thin film will be detected and recorded by a camera, fiber optic, and four‐point probe. Finally, the data are automatically inputted into ChemOS using the Phoenics global Bayesian optimization algorithm to analyze and design iterative experiments that guide composition ratio setting in the precursor solutions. After multiple iterations, excellent‐performance thin film materials will be selected. Ada takes about 21 min to synthesize, characterize, and plans in one experimental cycle for rapid ingredient screening. An open‐source Python software with strong compatibility is used to compile programs for a new experiment in the self‐driving laboratory.

The metal halide perovskite is a potential material for solar cells to increase efficiency. However, the performance of perovskite solar cells (PSCs) is curtailed due to nonradiative recombination. Passivation coating impedes PSCs’ rapid halide‐ion migration and chemical reactions with water, oxygen, and light. Xu et al.^[^ ^174^ ^]^ addressed the challenges of insufficient defect passivation by previous pseudohalide (PH) anions, which resulted in undesired deep impurity states. To combat this, the study utilized a ML workflow to accelerate the discovery of effective PH anions that could prevent lattice distortion and enhance attachment to the perovskite surface (Figure 16b). First, they screened PH anions from the PubChem database based on six screening tiers, and 168 candidates were selected for surface binding energy calculations including two calculation methods. Gaussian calculations and ML investigated the reason for the molecular structure of PH anions regulating their interaction strength with the perovskite surface. DFT calculations were performed for surface band edge electronic structures and defect formation energy calculations. 24 candidates were confirmed and further verified by experiments. Finally, 15 PH cations as passivation coating were found to be able to improve power‐conversion efficiency. This study combining theoretical calculation and ML provides guidance for the future design of optoelectronic materials.

#### 4.6.3 Cathode Coatings for Battery

**Figure 17:**
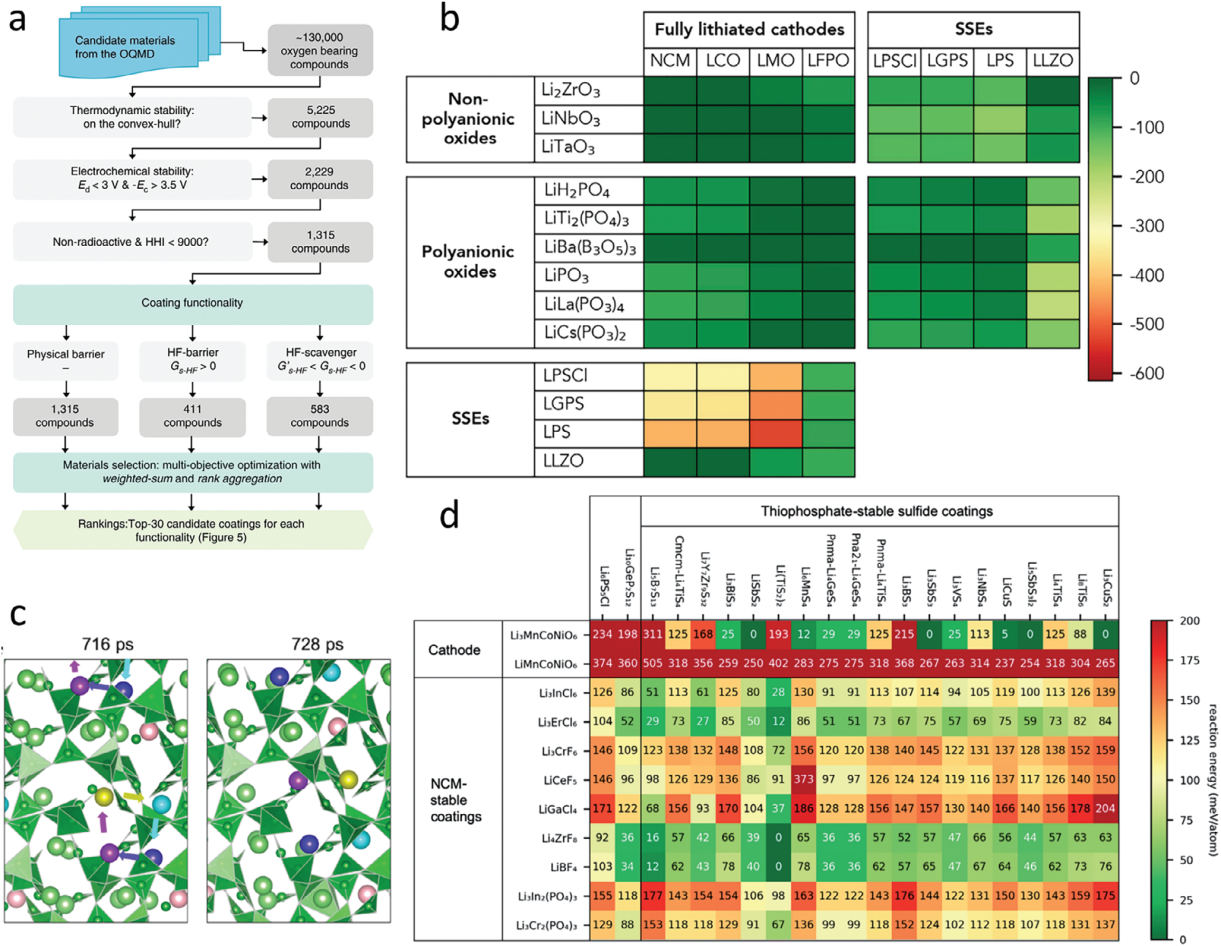
a) The flowchart of designing cathode coatings for Li‐ion battery by high‐throughput DFT calculation framework. Reproduced under the terms of the CC‐BY license.^[^ ^87^ ^]^ Copyright 2016, the Authors, published by Springer Nature. b) Chemical reaction energy Δ*E*_rxt_ with SSEs/coating and fully lithiated cathodes/coating and fully lithiated cathodes/SSEs. Dark green color means low chemical activity and vice versa. Reproduced with permission.^[^ ^178^ ^]^ Copyright 2019, Elsevier. c) The example of LOTF‐MD. The green tetrahedra and trigonal planar geometry is B*─*O bond, Li‐ion is sphere, and migrating Li‐ions are represented by different colored spheres and locally vibrating ones by green spheres. Reproduced with permission.^[^ ^179^ ^]^ Copyright 2020, American Chemical Society. d) Heat map of reaction energies at possible solid‐state battery interfaces. Reproduced with permission.^[^ ^180^ ^]^ Copyright 2021, Royal Society of Chemistry.

Li‐ion batteries are currently the most widely energy storage device, benefiting from their advantages of high energy density, high conversion efficiency, and fast response speed. However, users are criticizing the service life of Li‐ion batteries declining too fast, especially in mobile phones. At present, there is no good revolutionary technology to replace Li‐ion batteries, so that, methods for improvements must be studied. The important reasons for the decline in battery life are the dissolution of the redox‐active transition metal ions into the electrolyte, instability against irreversible phase transformations, the dissolution of the cathode, etc. Protective coating, such as Al_2_O_3_, MgO, ZnO, etc.,^[^ ^175^, ^176^, ^177^ ^]^ on the cathode surface, can isolate the cathode and electrolyte and effectively alleviate the occurrence of corrosion. But considering the complex reactions between the cathode, coating, and electrolyte, it is difficult to further select the appropriate multivariate. As mentioned previously, Aykol et al.^[^ ^87^ ^]^ proposed a strategy for rapid screening of ternary and beyond compounds by high‐throughput DFT calculations‐based framework to save the trouble of trial‐and‐error experimentation. The schematic of the method is shown in **Figure** **17**a. The researchers first screened the candidate materials by the criteria of thermodynamic stability for synthesizing experimentally, electrochemical stability for keeping intact in the battery, and nonradioactive & Herfindahl–Hirschman index (HHI) for commercial feasibility analysis from the OQMD. Then, they categorized the candidate materials into three types based on the ability of the materials used as a coating to react with HF: physical barrier for degradation mechanism little related to HF, HF‐barrier for protective coating, and HF‐scavenger for coating as a sacrificial anode. To balance multiple performance indicators for materials, the best candidate materials were screened by multiobjective optimization with the weighted‐sum and rank aggregation. The high similarity of the candidate materials predicted by the weighted sum and rank aggregation indicated the reliability of this work. The functional coatings predicted in this work significantly reduce the cost of materials exploration and aid in battery development. However, there are many factors affecting the performance of battery coatings. Screening by a few important ones could be acceptable, and optimal coatings can be better screened if more factors are considered. Xiao et al.^[^ ^178^ ^]^ further developed the strategy Aykol et al.^[^ ^87^ ^]^ proposed by considering whether the coating could be stable with both the cathode and the solid‐state electrolyte (SSE) and ionic conductivity for coating performance to screen the cathode coating material for the solid‐state batteries (SSBs) from the ICSD and those generated by applying data‐mined chemical substitutions. The main difference between the two is that the DFT calculations calculate the chemical stability screening of the latter. In addition, this work considered more reactivity between the fully lithiated cathodes and the coating, as well as the inorganic solid‐state electrolytes and the coating (Figure 17b).

As traditional screening methods, thermal and DFT calculations require considerable computational resources. The researchers are badly in need of a new approach that is economical to screen cathode coating material by theoretical analysis. Wang et al.^[^ ^179^ ^]^ combined on‐the‐fly ML and MD simulations to calculate the Li‐ion conduction in any crystalline material containing Li that is more efficient than the pure ab initio molecular dynamics (AIMD). A diagram of the LOTF‐MD (“learning on the fly”‐ML) process is shown in Figure 17c. The methodology is briefly described below. First, the on‐the‐fly ML provided interatomic potentials trained by AIMD data, and the cost depended on the number of data points. Second, the mean squared displacement (MSD) of Li‐ion less than 3 Å^2^ of materials was stripped away to avoid the room temperature Li‐ion nonconductors. Third, LOTF‐MD calculated the Li‐ion mobility in materials at different temperatures. The total mean squared displacement (TMSD) more fabulous than 2000 Å^2^ ensured that the diffusion values of Li‐ion had a sufficiently high rate at room temperature. Finally, the data was used to obtain the activation energy (*E*_a_) and room temperature conductivity related to room‐temperature tracer diffusivity (D (300K)) by fitting the Arrhenius function. This method eliminates the hassle of unavailable interatomic potentials, reduces cost dramatically compared to pure AIMD, and could be used to calculate other materials. They recently applied this method to screen for double‐layer cathode coatings in all‐solid‐state batteries.^[^ ^180^ ^]^ The process of this work is similar to the previous one. First, the Li‐containing compounds with a Li content greater than 10%, nonradioactive, and thermodynamic stability (energy above hull 30≤meV) were screened for the next step. Second, the interfacial reaction energies set to less than 4 of candidate materials with the thiophosphate electrolyte or cathode NCM/discharged NCM were calculated by AIMD. Finally, *E*_a_ was obtained by the LOTF‐MD to determine the final coating materials for the solid electrolyte and cathode (Figure 17d).

### 4.7 Advantages of Data‐Driven Approaches in Functional Coatings Development

**Figure 18:**
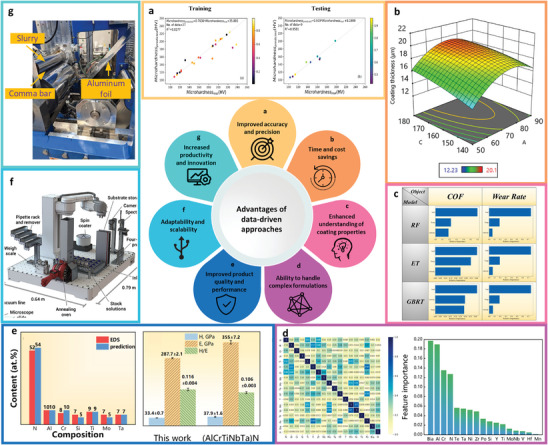
Benefits of applying a data‐driven approach to functional coatings. a) Improving the accuracy of predicting the microhardness of Ni‐P‐TiO_2_ composite coating by ML. Reproduced with permission.^[^ ^154^ ^]^ Copyright 2023, Elsevier. b) Effect of the electric potential and distance between the nozzle tip to the substrate on coating thickness. Reproduced with permission.^[^ ^84^ ^]^ Copyright 2021, Elsevier. c) Prioritizing factors affecting the coefficient of friction and wear rate aids understanding of the friction mechanism by different ML models. Reproduced with permission.^[^ ^181^ ^]^ Copyright 2023, Elsevier. d) Pearson correlation map importance ranking of features. Reproduced with permission.^[^ ^152^ ^]^ Copyright 2022, Elsevier. e) The predicted composition of coatings and their performance compared with (AlCrTiNbTa)N. Reproduced with permission.^[^ ^153^ ^]^ Copyright 2023, Elsevier. f) The Ada self‐driving laboratory. Reproduced under the terms of CC‐BY license.^[^ ^173^ ^]^ Copyright 2020, the Authors, pulished by American Association for the Advancement of Science. g) ML to handle data from the production process improves the productivity and quality of battery coatings. Reproduced with permission.^[^ ^182^ ^]^ Copyright 2022, Elsevier.

Data‐driven approaches offer numerous advantages in functional coatings development, including improved accuracy, time and cost savings, enhanced understanding of properties, increased productivity and innovation, improved product quality and performance, ability to handle complexity, and adaptability. These benefits make data‐driven approaches valuable tools in the formulation and optimization of functional coatings. **Figure** 18 presents examples of the advantages of the data‐driven approach, which will be described in the following sections.

#### 4.7.1 Improved Accuracy and Precision

Data‐driven approaches use large amounts of data to develop functional coatings, leading to improved accuracy and precision in predicting coating performance. This reduces the need for trial‐and‐error experimentation. For example, the coating performance can be modeled and optimized based on recorded datasets by ML to improve accuracy and precision. Shozib et al.^[^ ^154^ ^]^ developed ET model to accurately test the microhardness of the Ni‐P‐TiO_2_ coating (Figure 18a) because the experimental detection of microhardness was affected by many factors.

#### 4.7.2 Time and Cost Savings

By using data‐driven approaches, researchers can significantly reduce the time and cost of traditional trial‐and‐error methods. Data‐driven models can quickly analyze and predict coating performance, allowing researchers to optimize formulations in a shorter period. For instance, the preparation process of the coating contains many parameters, such as applied electrical potential, air pressure, substrate distance, flow rate, coating time, and so on. The coatings need to meet the parameters of achievable coating thickness, bonding strength, deposition efficiency, and so on. Paturi et al.^[^ ^84^ ^]^ applied ML to comprehend the complex parameters and establish the relationship between coating performance and their operational parameters (Figure 18b). The results presented a high prediction capability of electric potential, powder feed pressure, and distance between the nozzle tip to the substrate on coating thickness. Thus, it demonstrates that the ML can endow a precise estimate of coating thickness as well as curtailing time‐consuming and expensive electrostatic spray deposition experimental investigations. MacLeod et al.^[^ ^173^ ^]^ developed a self‐driving laboratory to take less than 21 min for an experimental cycle, significantly reducing research costs.

#### 4.7.3 Enhanced Understanding of Coating Properties

Data‐driven approaches provide insights into the relationships between different variables and coating properties. This deeper understanding allows researchers to identify key factors that influence coating performance and optimize formulations accordingly. For example, Levämäki et al.^[^ ^74^ ^]^ developed a method that combined ML and DFT calculations to discover the relationship between the disorder nitride coating and ordered nitride coating. Luo et al.^[^ ^183^ ^]^ summarized the law of the data of multicomponent rare‐earth silicide calculated by DFT. They found that the phase structure of multicomponent rare‐earth silicide was related to the related to the mean ($\bar{r}$) and mean square (σ_r_) of the rare‐earth elements. Further thermodynamic research has found that $\bar{r}$ and σ_r_ affect the conformational entropy, which determines the structure of the coatings (Figure 18c).

#### 4.7.4 Ability to Handle Complex Formulations

Functional coatings often involve complex formulations and require a balance between multiple properties. Data‐driven approaches can handle the complexity of these formulations and optimize coating performance across various properties simultaneously. Hao et al.^[^ ^149^ ^]^ used ML to develop a model to describe the oxidation and ablative properties of ultrahigh temperature ceramic coatings. 22 influencing factors were considered, and Pearson correlation map of features and importance ranking were used to derive each factor's priority in affecting the coatings' performance (Figure 18d). The approach gives the researchers the ability to quantitatively analyze the effects on the oxidative and ablative properties of a coating, allowing us to quickly identify the most critical indicators from a complex set of factors. Xu et al.^[^ ^152^ ^]^ also used ML to analyze the effect of multivariate on the properties of high entropy nitride coatings (Figure 18d). The influence of dozens of variables on coating performance is clear at a glance, and the direction of improving coating performance is available.

#### 4.7.5 Improved Coating's Quality and Performance

Researchers can optimize coating formulations using data‐driven approaches to meet specific performance criteria. This leads to improved product quality and performance, resulting in coatings that are more durable, resistant to wear and tear, or possess other desired properties. The multiple elemental variables make high‐entropy nitride coatings difficult to analyze for getting the optimal product quality and performance. Xu et al.^[^ ^152^ ^]^ prepared high‐entropy coating ((AlCrNbTaTi)N) with hardness 9% higher than the best quinary system through the ML and high‐throughput experiment (Figure 12c). Their team further screened a new composition coating of (AlCrTiMoTa)N with a hardness of 33.4 GPa and modulus of 287.7 GPa through ML and multiobjective optimization from the database of high‐entropy ceramic coating (Figure 18e).^[^ ^153^ ^]^

#### 4.7.6 Adaptability and Scalability

Data‐driven approaches can be easily adapted and applied to different coating systems and substrates. This scalability allows researchers to develop functional coatings for various applications, ranging from electronics to automotive and aerospace industries. MacLeod et al.^[^ ^173^ ^]^ developed a self‐driving laboratory to discover organic and inorganic materials relevant to materials sciences and clean energy technologies. (Figure 18f). The software supports Python, making it highly convenient for other researchers to modify the automated laboratory for their needs.

#### 4.7.7 Increased Productivity and Innovation

Data‐driven approaches enable researchers to screen and evaluate a wide range of formulation options quickly, leading to faster development of new functional coatings. This increased productivity promotes innovation in the field of coating development. Although data on industrial circulation can be collected by monitoring equipment and as a reference for optimizing industrial circulation, several factors affect product quality and production efficiency. A practical method is required for analysis. Liu et al.^[^ ^182^ ^]^ applied ML to collect data from real industrial circulation to build a model (Figure 18g). This model identifies the information on the coating mass, thickness, and porosity affecting the battery capacity and quantitatively deals with the effect of these three factors. The method directly influences manufacturing parameters' influence on battery coating performance, which can significantly facilitate engineers to improve production quality and innovation. Kolesnikov et al.^[^ ^184^ ^]^ combined finite element modeling and ML to optimize the parameters of double‐layer coating to design optimal multilayer coatings. Finite element modeling was used to simulate the microhardness of double‐layer coating, and ML was used to prepare the coating with a target hardness. This method dramatically improves the efficiency of conducting experiments by adjusting parameters one by one.

## 5 Challenges and Future Outlook

Data‐driven approaches offer a promising avenue for functional coatings development, providing researchers with powerful tools to accelerate the materials discovery process, improve the accuracy and reliability of prediction models, and enhance our knowledge of materials properties and performance. They have the potential to revolutionize the field of functional coatings by enabling researchers to discover new materials with exceptional properties in an efficient and systematic way. In this section, challenges of this field and some possible future directions are summarized.

### 5.1 Challenges of Data‐Driven Approaches in Functional Coatings Development

Although data‐driven materials discovery approaches in functional coatings have great potential, they also have challenges that need to be addressed.

#### 5.1.1 Limited Availability of High‐Quality Data

The success of data‐driven materials discovery largely depends on the quality of data used to train algorithms. Collecting high‐quality data can be challenging and expensive, especially for complex systems like functional coatings. It is preferable to obtain data from accredited database and reputable academic journals. Moreover, it is necessary to scrutinize the data in order to establish trust in the data identified, rather than establishing and applying a general quality assessment standard to distinct data sources.

#### 5.1.2 Lack of Standardization in Data Collection and Management

There is currently no standardized protocol for data collection and management in materials science. This can lead to inconsistencies in data and make it difficult to compare results across different studies. One of the hardest challenges associated with the standardization of data collection and management is overcoming the technological barrier in standardization of collection, aggregation and storage of data in multiple types, including structured data, unstructured data such as plain texts, images and videos. The standardization of data collection and management will guarantee the quality of the data collected as well as implement all relevant data sharing.

#### 5.1.3 Unpredictable Factors Affecting the Performance of Coatings

The performance of functional coatings can be affected by various factors, such as environmental conditions, manufacturing processes, and substrate properties. It can be challenging to account for all these factors in the development of data‐driven models. Since it is difficult to obtain a high‐quality dataset, a comprehensive dataset covering a variety of factors that impact performance of functional coatings should be used to improve the accuracy of the data‐driven models.

#### 5.1.4 Uncertainty in Predicted Results

The predictions made by data‐driven models have some level of uncertainty associated with them, which can make it difficult to confidently predict the behavior of functional coatings. Uncertainty of predictions due to data‐driven models should be quantified, represented and managed. Further, new prediction algorithms with good robustness, reliability and applicability issues should be developed to quantify uncertainty and improve accuracy of estimates, ensuring good prediction performances.

### 5.2 Prospects of Data‐Driven Approaches in Functional Coatings R&D

As materials scientists, it is essential for us to understand, embrace, and utilize data‐driven methods to signal the way forward and maximize the quality, cost‐effectiveness, and coverage of our care. We have to jump out of the comfort zone of those commonplace activities and broaden our horizons.

#### 5.2.1 Expansion of the Studied Materials Scope

Traditional coating materials mainly focus on one specific conventional need that is the surface protection of base materials. Currently, many data‐driven researches in this field are centered on traditional systems such as metals, ceramics, and polymers. As mentioned hereinbefore, any designed coating will face a complex environment during service in more and more modern applications scenarios, which is dictated by its intrinsic properties and must satisfy some specified requirements. This interaction can drastically limit the coating's lifetime or even change its properties, thus raising an increasingly demand in expanding research to include advanced functional coating materials such as 2D sheet material and bio‐based materials.

#### 5.2.2 Collaboration Between Experimentalists and Theorists

Experimental investigations are often used to study causal relationships, one or more independent variables are usually designed and their effect are measured and recorded. The process may be both costly and time‐consuming, particularly when dealing with complex coating systems, and the scientific laws and hidden mechanisms may lack of deep understandings to some extent. From the perspective of theoretical calculations, the above concerns can be handled, while the effectiveness and practicability are eager to be evidenced. As the challenges in materials science increase in scope and interdisciplinarity, collaborations, particularly among experimentalists and theorists, have become increasingly important in dealing with data‐driven affairs. A closer working relationship between experimental investigators and computational researchers can lead to more accurate predictions of coating behavior and better customization of desirable properties.

#### 5.2.3 Development of Database Management Technology for Information Systems

In recent years, booming advancements in hardware and software have contributed to the amelioration and emergence of various workflow paradigms for data management from experiments and computational simulations. The improvement of efficiency, reliability, and scalability of collecting, retrieving, storing, processing, and controlling data is and will be an important branch of information systems in data‐driven process. A novel distributed database management system are needed for different types data handling and coupling with any material computational software to improve the effectiveness of data‐driven materials discovery and better prediction of the coating service behavior.

#### 5.2.4 Improvement of Machine Learning Algorithms for Advanced AI

ML algorithms can learn and make predictions or decisions from data without being explicitly programmed, which is a key research foundation of advanced AI systems. Improving ML algorithms for advanced AI should pay attention to several approaches and techniques, such as model architecture, optimization algorithms, data augmentation, transfer learning, explainability, and Interpretability, and so on. Constructing a general machine learning model framework that integrates transfer learning and universal descriptors from functional coatings can enhance model's generalization capabilities and help to accelerate the process of functional coating development by identifying materials with desirable properties quickly and accurately. Study and innovation in these areas will contribute to the development of more powerful and intelligent AI systems for future data‐driven materials discovery.

#### 5.2.5 Integration of Sustainability and Environmental Impact Assessments

Functional coatings are normally fabricated on the surface of components in high‐tech industrial applications to provide desirable surface properties, which directly contact with human body or surrounding environments. Future research should pay close attention to the sustainability and environmental impact of new coatings developed through data‐driven materials discovery. This will help ensure that the materials developed are not only functional but also environmentally sustainable. In addition, sustainability and environmental impact are also important considerations in advanced technology and AI system. Thus, it is important to exploring and implementing strategies, such as improving time‐efficient and energy‐efficient algorithms, designing environmentally friendly computing systems, developing in situ environmental monitoring system and assessment, and emphasizing knowledge opening and sharing, that promote the development of sustainable and eco‐friendly data‐driven approaches in functional coatings development.

## 6 Conclusions

Data‐driven approaches have emerged as a prominent tool for accelerating the discovery of novel functional coatings. This review paper highlights the potential of data‐driven approaches in materials discovery and provides insights into the future of functional coatings research.

- Data‐driven materials discovery plays a crucial role in the development of functional coatings because it can help identify and optimize the properties of the materials needed for specific applications. Traditional trial‐and‐error methods can take a long time and may not produce the results needed for a particular application.
- Using data‐driven approaches, such as high‐throughput experimentation, computational modeling, ML, etc., researchers can quickly screen large numbers of materials and predict their properties before physical testing. These approaches accelerate the discovery process and supports the design of new and improved coatings tailored to specific needs.
- Moreover, data‐driven materials discovery allows for the combination of compositions, processes, and components that may have not been considered otherwise, opening up new horizons and possibilities for the development of functional coatings. Ultimately, this interdisciplinary methodology can lead to more efficient and effective coatings with longer lifetimes, higher performance, and even in situ self‐adaption in various applications.
- With the growing demand for coatings that exhibit advanced functionalities, such as anticorrosion, antifouling, and self‐healing, data‐driven approaches will play a critical role in accelerating the pace of materials discovery and innovation. These approaches have the potential to revolutionize the field of functional coating development and application. Therefore, it is critical for researchers in the field to embrace data‐driven approaches and techniques to overcome the limitations of traditional trial‐and‐error‐based approaches and develop high‐performance coatings that meet the ever‐evolving demands of modern industries. However, despite the promises, it is important to keep in mind that these approaches are still in their early stages of development and we must continue to validate these techniques against experimental data.

## Conflict of Interest

The authors declare no conflict of interest.
